# RNA binding induces an allosteric switch in Cyp33 to repress MLL1-mediated transcription

**DOI:** 10.1126/sciadv.adf5330

**Published:** 2023-04-19

**Authors:** Markus Blatter, Charlotte Meylan, Antoine Cléry, Roberto Giambruno, Yaroslav Nikolaev, Michel Heidecker, Jessica Arvindbhai Solanki, Manuel O. Diaz, Davide Gabellini, Frédéric H.-T. Allain

**Affiliations:** ^1^ Department of Biology, Institute of Biochemistry, ETH Zurich, 8093 Zurich, Switzerland.; ^2^ Gene Expression and Muscular Dystrophy Unit, Division of Genetics and Cell Biology, IRCCS San Raffaele Scientific Institute, Milan 20132, Italy.; ^3^ Department of Microbiology and Immunology, Stritch School of Medicine, Loyola University of Chicago Medical Center, University of Chicago, Chicago, IL, USA.

## Abstract

Mixed-lineage leukemia 1 (MLL1) is a transcription activator of the HOX family, which binds to specific epigenetic marks on histone H3 through its third plant homeodomain (PHD3) domain. Through an unknown mechanism, MLL1 activity is repressed by cyclophilin 33 (Cyp33), which binds to MLL1 PHD3. We determined solution structures of Cyp33 RNA recognition motif (RRM) free, bound to RNA, to MLL1 PHD3, and to both MLL1 and the histone H3 lysine N6-trimethylated. We found that a conserved α helix, amino-terminal to the RRM domain, adopts three different positions facilitating a cascade of binding events. These conformational changes are triggered by Cyp33 RNA binding and ultimately lead to MLL1 release from the histone mark. Together, our mechanistic findings rationalize how Cyp33 binding to MLL1 can switch chromatin to a transcriptional repressive state triggered by RNA binding as a negative feedback loop.

## Teaser

A three-position alpha-helix of Cyp33 acts as an allosteric switch induced by RNA binding to repress MLL1 mediated transcription.

## INTRODUCTION

**Fig. 1.**
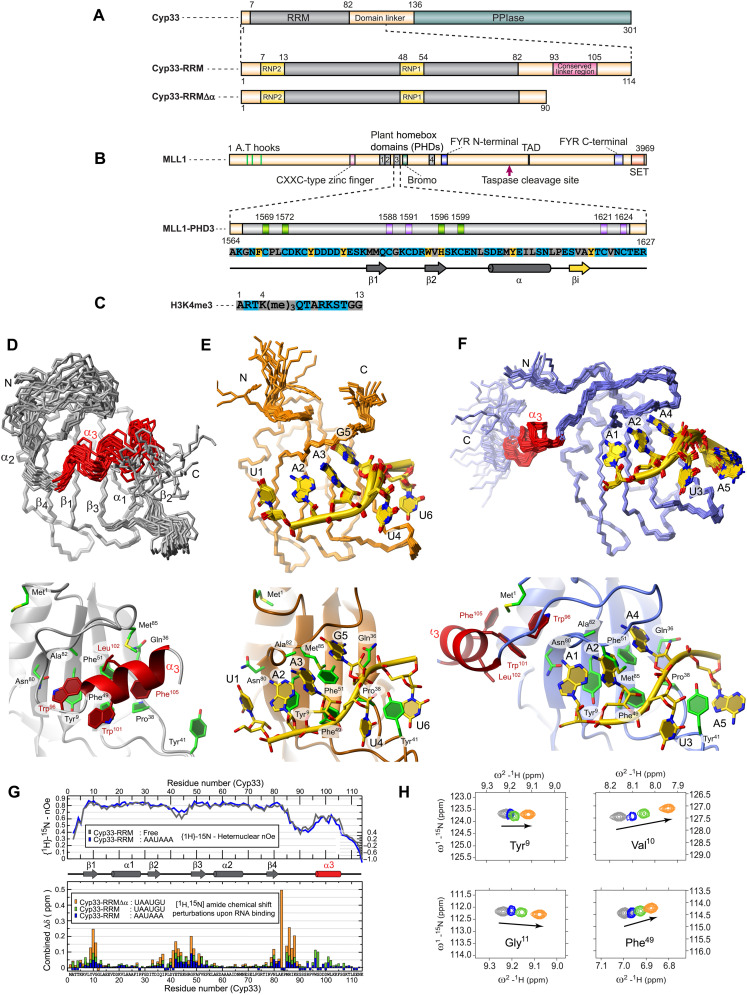
Cyp33 in free form and bound to RNA. (**A**) Cyclophilin 33 (Cyp33) with secondary structure elements of Cyp33 RNA recognition motif (RRM). (**B**) Mixed-lineage leukemia factor (MLL1). TAD, transactivation domain. (**C**) Histone H3 lysine N6-trimethylated (H3K4me3). (**D**) Top panel: Twenty energy lowest conformers in backbone representation of Cyp33 RRM. Bottom: most representative conformer in cartoon and stick representation. (**E**) Top panel: Twenty energy lowest conformers in backbone representation of Cyp33 RRM bound to UAAUGU RNA. Bottom panel: Most representative conformer in cartoon and stick representation. (**F**) Top panel: Twenty energy lowest conformers in backbone representation of Cyp33 RRM bound to AAUAAA RNA. Bottom panel: Most representative conformer in cartoon and stick representation. Color key: Cyp33 in free state (gray) bound to AAUAAA RNA (blue) and bound to UAAUGU RNA (green and orange); third helix (α3) is colored red. (**G**) Top panel: Heteronuclear nOe and perturbation plots of Cyp33 RRM free and bound to AAUAAA RNA. Bottom panel: [^1^H,^15^N] combined chemical shift difference plot of backbone amides for complexation at equimolar stoichiometries. Secondary structure is indicated with arrows (β strands) or tubes (α helices). The third α helix is illustrated in red. Unassigned backbone amides including prolines are displayed with a negative value. (**H**) Overlay of [^15^N-^1^H]-HSQC spectra of selected resonances of the free state Cyp33 RRM (gray cross-peaks), Cyp33 RRM bound to AAUAAA RNA (blue cross-peaks) or UAAUGUCG RNA (green cross-peaks), and UAAUGUCG RNA bound to Cyp33 RRMΔα (orange cross-peaks). ppm, parts per million.

Cyclophilin 33 (Cyp33) is a 33-kDa protein with a C-terminal cyclophilin domain (*1*, *2*) separated from an N-terminal RNA recognition motif (RRM) (*3*) by a partially conserved linker (Fig. 1A) (*4*, *5*). This conserved region is not part of the canonical RRM fold, raising the question of its potential role in association with interacting partners. The RRM of Cyp33 can bind not only AU-rich RNAs (*6*, *7*) but also the protein mixed-lineage leukemia 1 (MLL1) by interacting with its third plant homeodomain (PHD3) (*5*, *8*).

MLL1 (430 kDa) is a positive regulator of gene transcription in early development and hematopoiesis (*9*–*11*). The protein contains a bromodomain (BRD) and four PHDs (Fig. 1B). The third MLL1 PHD specifically interacts with methylated lysine marks (*12*, *13*). Furthermore, MLL1 has a methyltransferase activity and thereby is not only an epigenetic reader but also a writer. This activity was shown to be stimulated by the three conserved factors WD Repeat Domain 5 (WDR5), Retinoblastoma-binding protein 5 (RBBP5), and Set1/Ash2 histone methyltransferase complex subunit ASH2 (ASH2L) (*14*). Proteolytic cleavage by Taspase1 divides MLL1 into two subunits, named MLL1^N^ and MLL1^C^, which associate to form a holocomplex (*15*, *16*). Following AT-hooks and nuclear localization signals, MLL1^N^ harbors a CXXC domain, a repression domain, the first three PHDs (PHD1 to PHD3) and an adjacent BRD, followed by an extended atypical PHD (PHD4) and the FYRN domain. The methyltransferase active site, the SET domain, is located in the MLL1^C^ subunit, preceded by the FYRC domain and a transactivation domain (TAD) (Fig. 1B) (*17**)*.

Human MLL1 congenital mutations result in developmental abnormalities known as the Wiedemann-Steiner syndrome (*18*). In addition, MLL1 is an oncogene associated with leukemia (*19*–*21*). Chromosomal translocations resulting in the translation of fusion proteins between MLL1 and one of more than 90 different partners are found in leukemia cases, called MLL, where the same cells have both (mixed) myeloid and lymphoid traits (*19*, *22*–*25*). All MLL1 fusions lack the highly conserved three PHD cassettes, which is required for the fusion protein to become a constitutive transactivator. Reinsertion of the PHD3 blocks hematopoietic progenitor malignant transformation, supporting a key role for PHD3 in regulating MLL1 function (*26*, *27*).

The PHD3 of MLL1 is a 7.5-kDa domain that coordinates two zinc ions in a cross-brace scheme, including two large loops, a short β-turn and an 8–amino acid–long α helix (*28*). The PHD3 interacts with both Cyp33 RRM and Lys^4^ methyl marks on the histone H3 protein tail (H3K4me) (*4*, *12*, *13*, *28*). Interaction with the RRM β sheet is mediated by the α-helical part of MLL1 PHD3, while the first loop and the MLL1 PHD3’s β-turn specifically recognize the trimethyl marks of histone H3 lysine N6-trimethylated (H3K4me3) within an aromatic amino acid box (*13*, *28*). The atypical BRD does not interact with acetyl-lysines but rather regulates the association between Cyp33 and MLL1 by steric hindrance of the PHD3’s binding surface for Cyp33 (*29*). The SET domain of MLL1, as a component of the COMbinatorial Pathway ASSembly (COMPASS) complex, is involved in catalysis of H3K4 di- and trimethylation (H3K4me2 and H3K4me3) (*30*–*32*). Interaction with Cyp33 transforms MLL1 to a transcriptional repressor, affecting the expression of a large number of genes (*4*, *8*, *28*). In addition, after overexpression of CYP33 in human embryonic kidney (HEK) 293T cells, KDM5A and KDM5B are recruited to the MLL1 target gene promoters and H3K4 is demethylated (*33*).

The β sheet surface of Cyp33 RRM was shown to interact with both RNA and the PHD3 of MLL1 (*4*, *13*, *28*), suggesting mutual exclusion and competitive binding. Whereas the poly-A signal AAUAAA was proposed as RNA target in the context of Cyp33’s function in protein folding (*7*, *34*), a recent study identified AAUAAUAA as a systematic evolution of ligands by exponential enrichment (SELEX) consensus motif for this protein (*35*). Such a motif can be found in multiple copies in the long intergenic noncoding RNAs (lincRNAs) *NC3* and *NC4* located between *HOXC8* and *HOXC6*, which are both bound by MLL1 (*36*). Wang *et al*. (*13*) showed that Cyp33 uses its peptidylprolyl isomerase (PPIase) activity to isomerize MLL1 proline-1629 located between the BRD and PHD3 domains. This *cis*-*trans* isomerization acts as a switch by introducing a conformational change, which dissociates the BRD domain from PHD3 and allows the binding of Cyp33 RRM to MLL1 PHD3 domain. Binding of Cyp33 to MLL1 induces the recruitment of the histone deacetylase HDAC1 via the repression domain (*27*, *37*) and of histone demethylases of the JARID1 family known to remove H3K4 methyl marks (*33*). On the basis of these observations, it was proposed that binding of Cyp33 to RNA could prevent the transition of an H3K4me3-bound MLL1 transcriptional active state to a repressive state (*4*, *13*, *28*, *35*).

Here, we provide critical information on this regulatory system through the structures of Cyp33 RRM free, in complex with RNA, with MLL1 PHD3 and with MLL1 PHD3 bound to H3K4me3. They revealed a crucial role for the conserved C-terminal extension of the RRM, which folds into a third α helix extending the domain. This third α helix adopts three different positions relative to the RRM domain depending on the different Cyp33 binding partners and appears to function as an allosteric switch dictating the sequence of events leading to transcription repression by MLL1. Our results can now explain the previously unclear and controversial role of RNA binding by Cyp33 as well as the existence and role of a ternary complex between Cyp33, MLL1, and H3K4me3.

## RESULTS

### Solution structure of the Cyp33 RRM domain reveals the presence of an additional α3 helix

Cyp33 consists of an N-terminal RRM domain and a C-terminal cyclophilin domain separated by a partially conserved linker of unknown function (Fig. 1A). The cyclophilin domain has catalytic PPIase activity, and the RRM is primarily involved in the recruitment of binding partners. Because the C-terminal extremity of the RRM is well conserved and was previously proposed to be important for Cyp33 function (*5*), we cloned and expressed the human Cyp33 RRM with and without this C-terminal extension (Cyp33 RRM and Cyp33 RRM∆α; Fig. 1A). Both protein constructs in their free form gave well-dispersed nuclear magnetic resonance (NMR) spectra with sharp linewidth (fig. S1, C and D). Only parts of the second and the third β strand (β_2_β_3_-loop) and the C-terminal extension experienced some line broadening, indicating conformational exchange. Using 3112 nuclear Overhauser effect (nOe)-based distance restraints (table 1), we could determine a highly precise structure of the RRM core domain, whereas the C-terminal extension was less precise, reflecting some mobility as represented in the final structural ensemble (Fig. 1D).

The structure of the RRM is similar to the crystal structure determined previously (*13*). However, the conserved C-terminal sequence was missing in the x-ray structure. Unexpectedly, this C-terminal extremity of the RRM adopts an α helix structure (α3), which interacts with the RRM β sheet surface (Fig. 1D). Trp^101^, Leu^102^, and Phe^105^ of this α-helical region shields the hydrophobic patch of the RRM β sheet composed of the three exposed aromatic residues Tyr^9^ (RNP2), Phe^49^, and Phe^51^ (RNP1) together with Pro^38^ at the end of β_2_ (Fig. 1D). In this conformation, the canonical RNA binding surface of the RRM is occluded. Backbone ^15^N-[^1^H]-nOe experiments showed high flexibility between the end of the canonical RRM fold and the conserved linker sequence (residues 85 to 94) and moderate dynamics for the C-terminal α helix (residues 95 to 106) together with the β_2_β_3_-loop (Fig. 1G). In conclusion, the structure of Cyp33 RRM combined with the ^15^N-[^1^H]-nOe data and the broadening of Trp^101^, Leu^102^, and Phe^105^ (fig. S1C) suggests that, in the free form, α_3_ is loosely interacting with the RRM β sheet surface and predominantly oriented perpendicular to it.

### Solution structure of Cyp33 RRM bound to RNA shows a relocation of the α3 helix on the side of the RRM

Because α_3_ occludes the canonical binding site of Cyp33 RRM, its interaction with RNA could be compromised. Therefore, we investigated whether the binding of the RRM to RNA was still possible. We first performed NMR titrations using the mRNA poly-A signal sequence AAUAAA that was proposed to be targeted by Cyp33 (*6*). The chemical shift perturbations were very small, indicating a low binding affinity (Fig. 1, G and H). Nevertheless, the mapping of perturbed resonances on the protein sequence showed clustering in one continuous region of the structure, namely, the β sheet surface and parts of α3 (Fig. 1G). This suggested that the β sheet surface of the RRM binds to the RNA as shown previously (*4*). The observation of many intermolecular nOes between the RNA and the protein of this complex further supported this interaction (fig. S1E). We then decided to determine the solution structure of Cyp33 RRM bound to AAUAAA RNA. Sharp linewidths of the RRM and RNA were obtained by using 1.5 molar equivalents of AAUAAA in all samples (Fig. 1H), and many intermolecular nOes were detected (fig. S1E). However, they indicated that the RNA interacted with Cyp33 RRM in multiple registers (fig. S1E). Therefore, we decided to use another RNA target hoping to experience less conformational exchange.

Because SELEX data were not available at that time, we performed a SELEX experiment with Cyp33 and identified a YAAUNY RNA binding consensus sequence (Y and N are for pyrimidine and any nucleotide, respectively) (fig. S1, F and G) very close from the AAUAAUAA motif identified recently using the same method (*35*). This motif is found in multiple copies in the *NC3* and *NC4* lincRNA (fig. S1B), which are transcribed from the intergenic sequence of MLL1 target genes *HOXC8* and *HOXC6*. Consequently, we titrated Cyp33 RRM to UAAUGUCG that contains the consensus motif and two additional nucleotides. Similar chemical shift perturbations were observed for AAUAAA and UAAUGUCG, indicating a similar mode of interaction with the RRM for both sequences (Fig. 1, G and H). Although the amplitude of the chemical shift perturbations doubled with this latter RNA, the complex formation was still in fast exchange regime, indicating still a weak interaction. We hypothesized that this low RNA binding affinity might originate from the presence of the α3 helix that might occlude the canonical β sheet surface of the RRM. Therefore, we investigated RNA binding to the Cyp33 RRM lacking α3 (Cyp33 RRM∆α). NMR titration experiments of this shortened construct Cyp33 RRM∆α with UAAUGUCG showed much larger chemical shift changes (two- to fourfold larger) compared to Cyp33 RRM (Fig. 1, G and H). We could determine the structure of the complex observing a single register (Table 1). The structure revealed that all nucleotides of the SELEX consensus are bound but not the two additional nucleotides at the 3′ end (Fig. 1E and fig. S1H). A large network of intermolecular hydrogen bonds (fig. S1H) further supported this binding mode of the consensus sequence.

**Table 1.** Statistics from solution structures of Cyp33, MLL1, and histone H3 complexes. RRM, RNA recognition motif; MLL1, mixed-lineage leukemia 1; Cyp33, cyclophilin 33; PHD3, third plant homeodomain; NMR, nuclear magnetic resonance; H3K4me3, histone H3 lysine N6-trimethylated; RMSD, root mean square deviation; PDB, Protein Data Bank; BMRB, Biological Magnetic Resonance Bank.

	Cyp33-RRM*	Cyp33-RRM: AAUAAA^†^	Cyp33-RRMΔα: UAAUGUCG^‡^	Cyp33-RRM: MLL1-PHD3^§^	Cyp33-RRMΔα: MLL1-PHD3:H3K4me3^||^
**Completeness of ^1^H chem. shift assignm. (%)^¶^**	96.1	97.2	99.5	92.4	89.9
Cyp33 (%)	96.1	96.9	99.4	92.8	94.9
RNA or MLL1 (%)		100	100	91.6	86.9
H3K4me3 (%)					65.6
NMR restraints					
Distance restraints	3112	3780	3936	4548	3262
Cyp33 intramolecular	3112	3627	3668	3193	2362
Intraresidual	562	643	576	552	454
Sequential (|*i* − *j*| = 1)	860	942	896	807	630
Medium range (1 < |*i* − *j*| < 5)	654	831	779	733	497
Long range (|*i* − *j*| ≥ 5)	1036	1211	1417	1101	781
RNA or MLL1 intramolecular		42	132	1129	624
Intraresidual		36	98	246	175
Sequential (|*i* − *j*| = 1)		6	29	345	213
Medium range (1 < |*i* − *j*| < 5)		0	5	284	135
Long range (|*i* − *j*| ≥ 5)		0	0	254	101
H3K4me3 intramolecular					40
Intraresidual					31
Sequential (|*i* − *j*| = 1)					4
Medium range (1 < |*i* − *j*| < 5)					5
Cyp33:RNA or Cyp33:MLL1 intermolecular		111	136	226	162
MLL1:H3K4me3 intermolecular					74
Torsion angles^#^	0	60	8	176	0
Cyp33 backbone	0	54	0	122	0
MLL1 backbone				54	0
H3K4me3 backbone					0
RNA sugar pucker		6	8		
Energy statistics**					
Average distance constraint violations					
0.1–0.2 Å	15.0 ± 2.7	15.0 ± 2.7	15.0 ± 2.7	9.6 ± 2.0	24.4 ± 4.3
0.2–0.3 Å	0.8 ± 1.0	0.9 ± 1.0	0.1 ± 0.2	0.5 ± 0.5	4.0 ± 1.2
>0.3 A	0.6 ± 0.7	0.1 ± 0.2	0.0 ± 0.0	0.1 ± 0.4	0.2 ± 0.4
Maximal (Å)	0.27 ± 0.1	0.21 ± 0.03	0.16 ± 0.02	0.21 ± 0.06	0.28 ± 0.03
Average angle constraint violations					
<5°		22.6 ± 2.2	3.0 ± 0.0	19.6 ± 2.0	0.0 ± 0.0
>5°		0.0 ± 0.0	0.0 ± 0.0	0.0 ± 0.0	0.0 ± 0.0
Maximal (°)		1.44 ± 1.18	0.32 ± 0.03	0.64 ± 0.21	0.0 ± 0.0
Mean AMBER constr. viol. energy $\left(\frac{\mathrm{kcal}}{\mathrm{mol}}\right)$	21.3 ± 3.0	24.0 ± 2.6	11.1 ± 0.9	15.4 ± 1.9	23.1 ± 1.7
Distance $\left(\frac{\mathrm{kcal}}{\mathrm{mol}}\right)$	21.3 ± 3.0	21.3 ± 1.6	10.7 ± 0.9	15.3 ± 1.9	22.9 ± 1.7
Torsion $\left(\frac{\mathrm{kcal}}{\mathrm{mol}}\right)$		2.8 ± 2.2	0.4 ± 0.1	0.1 ± 0.1	0.2 ± 0.1
Mean AMBER energy $\left(\frac{\mathrm{kcal}}{\mathrm{mol}}\right)$	−3174 ± 12	−4229 ± 9	−4099 ± 10	−5141 ± 8	−5261 ± 19
Mean deviation from ideal covalent geometry					
Bond length (Å)	0.004 ± 0.000	0.004 ± 0.000	0.004 ± 0.000	0.004 ± 0.000	0.004 ± 0.000
Bond angle (°)	1.651 ± 0.018	1.758 ± 0.012	1.768 ± 0.014	1.624 ± 0.014	1.641 ± 0.018
Ramachandran plot statistics**^,††^					
Residues in most favored regions (%)	90.0 ± 2.1	82.5 ± 2.1	84.6 ± 1.6	88.2 ± 1.4	86.5 ± 1.9
Residues in additionally allowed regions (%)	10.0 ± 2.1	17.5 ± 2.1	15.4 ± 1.6	11.6 ± 1.4	13.0 ± 1.9
Residues in generously allowed regions (%)	0.0 ± 0.0	0.0 ± 0.0	0.0 ± 0.0	0.2 ± 0.4	0.4 ± 0.6
Residues in disallowed regions (%)	0.0 ± 0.0	0.0 ± 0.0	0.0 ± 0.0	0.0 ± 0.2	0.2 ± 0.4
RMSD to mean structure statistics**					
Cyp33					
Backbone atoms	0.18 ± 0.03	0.26 ± 0.05	0.12 ± 0.03	0.28 ± 0.04	0.20 ± 0.05
Heavy atoms	0.53 ± 0.08	0.52 ± 0.08	0.42 ± 0.08	0.59 ± 0.06	0.53 ± 0.04
RNA or MLL1					
Backbone atoms		0.34 ± 0.11	0.19 ± 0.05	0.50 ± 0.11	0.77 ± 0.17
Heavy atoms		0.47 ± 0.18	0.29 ± 0.05	0.84 ± 0.11	1.25 ± 0.20
H3K4me3					
Backbone atoms					0.38 ± 0.20
Heavy atoms					1.03 ± 0.22
All molecules					
Backbone atoms	0.18 ± 0.03	0.29 ± 0.05	0.15 ± 0.03	0.46 ± 0.06	0.63 ± 0.15
Heavy atoms	0.53 ± 0.08	0.54 ± 0.07	0.41 ± 0.07	0.76 ± 0.06	0.99 ± 0.15
**PDB code**	7ZEV	7ZEW	7ZEX	7ZEY	7ZEX
**BMRB code**	34724	34725	34726	34727	34728

*On the basis of the structured residue range of Cyp33-RRM: 5–38,47–84 (-3–114, chain A).

†On the basis of the structured residue range of Cyp33-RRM: 5–84,96–106 (-3-114, chain A); AAUAAA RNA: 1–5 (1–6, chain B).

‡On the basis of the structured residue range of Cyp33-RRMΔα: 5–84 (-3–90, chain A); UAAUGUCG RNA: 1–6 (1–8, chain B).

§On the basis of the structured residue range of Cyp33-RRM: 5–84,96–106 (-3–114, chain A); MLL1-PHD3: 1568–1625 (1564–1627, chain B).

||On the basis of the structured residue range of Cyp33-RRMΔα: 5–84 (-3–90, chain A); MLL1-PHD3: 1568–1625 (1564–1627, chain B); H3K4me3: 1–7 (1–13, chain C).

¶NMR observable ^1^H chemical shift assignment completeness determined by the CYANA command peakcheck.

#Protein backbone angles of structured residues determined by the program TALOS+. Sugar pucker angles based on coupling efficiency in homonuclear TOCSY.

**Statistics computed for the bundle of 20 energy best structure refined against the AMBER ff12SB force field.

††Ramachandran plot, as defined by the program PROCHECK (*62*).

Because the chemical shift perturbation mapping and directions of the shifts indicated a very similar binding of the RRM to the two RNAs tested, we compared the nOes in the spectra of the one register binding complex Cyp33 RRM∆α: UAAUGU to the ones of the multiple registers binding complex Cyp33 RRM: AAUAAA. The vast majority of these intermolecular nOes could be found among the most intense nOes arising from the different registers. Using this set of restraints, we could determine the structure of the Cyp33 RRM bound to AAUAAA, which included α3 (Fig. 1F). In this structure, α3 dislocates from the β sheet and relocalizes to form new contacts with the N-terminal extremity of the protein using the same hydrophobic residues (Trp^99^, Trp^101^, Leu^102^, and Phe^105^) as those used to interact with the β sheet surface in the free form (Fig. 1, D and F). Analysis of the two protein-RNA recognition interfaces of Cyp33 RRM bound to AAUAAA and Cyp33 RRM∆α bound to UAAUGU revealed a binding consensus for YAAURN (where Y is a pyrimidine, N is any nucleotide, and R is a purine; Fig. 1 and fig. S1H). This is in remarkable agreement with the SELEX consensus identified in this study (YAAUNY) and previously (AAUAAUAA) (*35*).

### RNA binding stimulates Cyp33 transcription repression activity

Our structure of Cyp33 bound to RNA revealed that the α3 helix had to be displaced from the β sheet surface to allow the binding of the RRM to RNA. Next, we investigated whether this RNA-driven unusual structural rearrangement had any relevance to Cyp33 function as a regulator of MLL1 activity. On the basis of our structural findings, we designed mutants for which the RNA binding affinity of Cyp33 should be decreased (K83A, RK86-88A, and KRK83-88A as single-, double-, and triple-mutant variants) and a W101A, L102A, and F105A triple mutant (Cyp33-WLF) for which the RNA binding affinity of the RRM should be enhanced (fig. S2A). The mutated lysines and arginine are involved in interactions of the RRM with the backbone and bases of the RNA (fig. S1H). Because K83 was also reported to be a ubiquitination site of Cyp33, we did not use this mutation for in vivo investigations and rather based our conclusions on the double-mutant Cyp33-RK86-88A. Conversely, the tryptophan, leucine, and phenylalanine do not contact RNA. They are the core residues in α3 responsible for the hydrophobic packing of the helix on the β sheet (fig. S2A). In good agreement with the expected effect of these mutations, the chemical shift perturbations on the β sheet induced by the WLF-mutated helix in the free protein are smaller than those of the wild type (WT) compared to Cyp33 RRMΔα, confirming that α3 interacts less efficiently with the β sheet of Cyp33 RRM in this mutant (fig. S2B). We then measured the affinities of all the Cyp33 mutants for UAAUGU RNA and for the MLL1 PHD3 domain (fig. S2, C and D). The binding affinities for PHD3 were measured by ITC and were unchanged for all the mutants [dissociation constant (*K*_d_) values around 7 μM; table S1], indicating that they only affect RNA binding. For the Cyp33-WLF mutant, the RNA binding affinity was measured by both isothermal titration calorimetry (ITC) and NMR, whereas for the Cyp33-WT and the other mutants affinities were measured only by NMR titrations. Cyp33-WT binds to UAAUGU RNA with a *K*_d_ of 300 μM. As we predicted, the α3 mutant Cyp33-WLF has a higher RNA binding affinity (*K*_d_ of about 70 μM), whereas the Cyp33-K, Cyp33-RK, and Cyp33-KRK mutants bind RNA with a much lower affinity (*K*_d_ values of 1.8 and 2.0 mM and higher than 10 mM, respectively) (table S1 and fig. S2C).

**Fig. 2.**
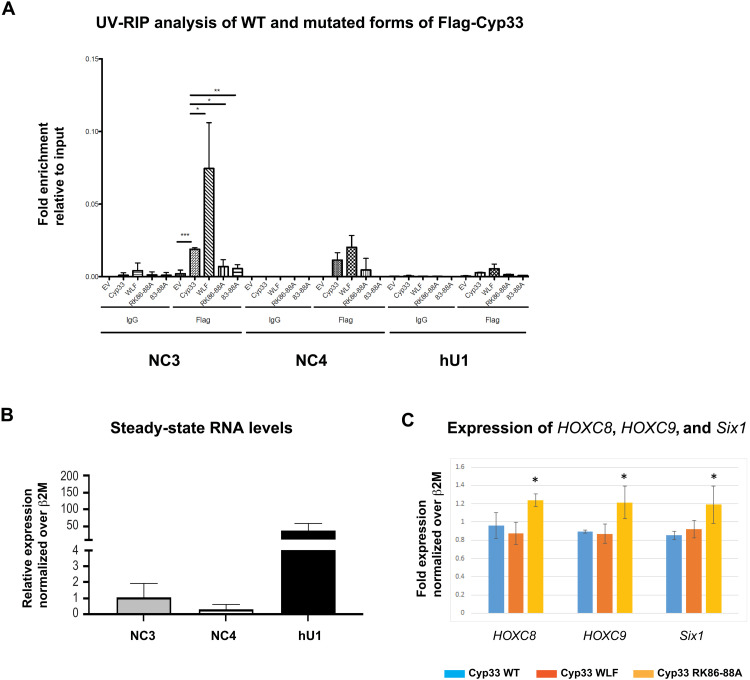
Cyp33 interacts with NC3 and NC4 and inhibits MLL1-mediated RNA transcription. (**A**) Ultraviolet (UV)–RNA immunoprecipitation (RIP) analyses of wild-type (WT) and mutated forms of Flag-Cyp33 overexpressed in human embryonic kidney (HEK) 293 cells. Immunoglobulin Gs (IgGs) were used as negative controls for the RIP. The data displayed in the figure are the average of three biological replicate experiments (*n* = 3) and are represented as fold enrichment over input material. Statistical analysis was performed using nonparametric two-tailed *t* test. *P < 0.05; **P < 0.01; ***P < 0.001. (**B**) Relative expression of NC3, NC4, and hU1 normalized over β2M. (**C**) Expression of HOXC8, HOXC9, and Six1 in HEK293T cells transfected with WT (in blue) and mutated versions (WLF 101–105 and RK86–88A in red and orange, respectively) of Cyp33 proteins after normalization over β2M. Values are expressed as fold change with respect to cells transfected with the empty vector (EV) control considered equal to 1. Statistical analysis was performed using nonparametric two-tailed *t* test. *P < 0.05.

To test whether these in vitro results would be functionally relevant, we performed ultraviolet (UV) cross-linking followed by RNA immunoprecipitation (UV-RIP), which uses an antibody to purify a specific protein and detects RNA bound to it, taking advantage of the fact that UV light only cross-links proteins and nucleic acids that are directly interacting. By UV-RIP, we evaluated the association of Flag-tagged Cyp33-WT and mutant proteins (WLF, RK86-88A, and KRK 83-88A) with *NC3* and *NC4* lincRNAs, which are located in the intergenic region of the MLL1 target genes *HOXC8* and *HOXC6* (fig. S1). Flag-Cyp33 proteins were expressed at similar levels after 24 hours (fig. S2G) and then immunoprecipitated. The level of associated RNAs (*NC3*, *NC4*, and *hU1* as a negative control) was estimated by reverse transcription quantitative polymerase chain reactions (RT-qPCRs). As shown in Fig. 2A, an interaction of Cyp33-WT with *NC3* and *NC4* RNAs was observed. In perfect agreement with the in vitro data, the WLF mutant interacted more efficiently with *NC3* and *NC4*, whereas a decrease in interactions was observed for the RK86-88A and KRK83-88A variants (Fig. 2A). These interactions are specific, as almost no binding was detected with *hU1*, despite the fact that this RNA is expressed at much higher levels compared to *NC3* and NC4 (Fig. 2, A and B).

We then tested the effect of Cyp33 WT and its RNA binding mutants on MLL1-mediated transcriptional control. To do so, we transfected HEK293 cells with WT or mutated versions of Cyp33 and investigated the expression of the MLL1-controlled genes *HOXC8*, *HOXC9*, and *Six1* (*28*, *36*) by RT-qPCR. Whereas the α3 mutant Cyp33-WLF did not affect transcription levels, the Cyp33-RK mutant increased transcription of all genes tested (Fig. 2C and fig. S2H) in line with the possibility that RNA binding by Cyp33 contributes to transcription repression. We next addressed the molecular basis at the origin of this effect.

We first investigated whether Cyp33 interaction with RNA could stimulate PPIase activity as previously suggested (*6*) using an enzyme-coupled catalytic PPIase activity assay with chymotrypsin and *N*-succinyl-Ala-Ala-Pro-Phe *p*-nitroanilide as substrates in combination with our highly specific Cyp33 mutants. By comparing the catalytic PPIase activity at different concentrations (5 to 40 nM) of Cyp33 free and bound to 200 μM UAAUGU RNA (the chosen excess leads to more than 50% bound form), we measured turnover numbers of 150.3 ± 13 s^−1^ for the ligand-free Cyp33 and 149.7 ± 12.4 s^−1^ for the RNA-bound Cyp33 (fig. S2I). Because the PPIase activity of Cyp33 was the same in the absence and presence of RNA, we conclude that this Cyp33-RNA interaction does not contribute to its PPIase activity. We next went on to investigate whether RNA binding by Cyp33 might affect the interaction of MLL1 with the H3K4me3 histone mark.

### Cyp33-RNA complex can release H3K4me3 from MLL1 PHD3

To study this, we first prepared a stoichiometric complex between Cyp33 full-length (FL) and the RNA UAAUGU (fig. S3A). Using NMR, we could observe this complex formation by observing changes in the chemical shift of U4 H6 or G5 H8 upon Cyp33 FL titration (fig. S3A). Next, we mixed this complex to the one formed between MLL1 PHD3 and an H3K4me3 peptide at a 1:1:1:1 stoichiometry, and NMR data were measured immediately and after 15 min. We could observe that the RNA peaks were then shifting back to a position close to the RNA-free form, indicating a release of the RNA from Cyp33 RRM. This result was somehow expected, since the β sheet surface of the domain involved in RNA binding was shown previously to also interact with MLL1 PHD3 (*4*). Their interaction with the β sheet is then mutually exclusive. Nevertheless, the RNA was apparently not completely released in a free form, since the chemical shifts were not identical at the beginning and at the end of the titration (blue and red curves in fig. S3A). We then wondered whether the released RNA could not be trapped by the histone H3 tail, as it contains four positively charged residues and no negatively charged one (Fig. 1C). We then decided to follow the chemical shift change of the intense NMR signal of the three methyl groups of K4me3 of the peptide in the presence of RNA. Unfortunately, a peak coming from a contaminant present in the synthesized RNA was overlapping with the characteristic peptide peak. We then had to transcribe an RNA containing four repeats of the synthesized RNA sequence to be able to transcribe and purify it. The use of an RNA containing multiple repeats was biologically relevant, as several copies of the motif bound by Cyp33 are found in NC3 and NC4 transcripts (fig. S1). In the presence of increasing amount of this RNA, we clearly observed a chemical shift perturbation of the peptide signal from 3.15 to 3.12 parts per million (ppm) (fig. S3B), showing that the H3K4me3 peptide interacts with this RNA. The binding could be quantified with a *K*_d_ of 30 μM measured with ITC (fig. S2F). Notably, the signal of the peptide observed upon addition of the MLL1-H3K4me3 (1:1) complex to a large excess of the Cyp33:RNA (1:1) complex shifted to the exact same position (3.12 ppm; fig. S3C), which was different from the chemical shift observed for the free peptide (3.15 ppm; fig. S3B). These data indicate that the peptide is binding to the released RNA upon dissociation from Cyp33 RRM. This result was clearly more unexpected than the release of RNA, since it was shown earlier that Cyp33 RRM, MLL1 PHD3, and H3K4me3 could form a trimolecular complex (*13*). However, the presence of RNA and its interaction with the peptide further stabilizes its release. This dissociation of the peptide from MLL1 PHD3 was further confirmed by the observation of chemical shift changes toward the free form of the PHD3 residues involved in the interaction with the H3K4me3 peptide (e.g., W231) (fig. S3D). To better understand this release of the histone tail upon Cyp33 binding, we went on to investigate structurally the final product, namely, the complex of Cyp33 RRM bound to MLL1 PHD3.

### Solution structure of Cyp33 RRM in complex with MLL1 PHD3 shows a relocation of the α3 helix parallel to the β sheet

**Fig. 3.**
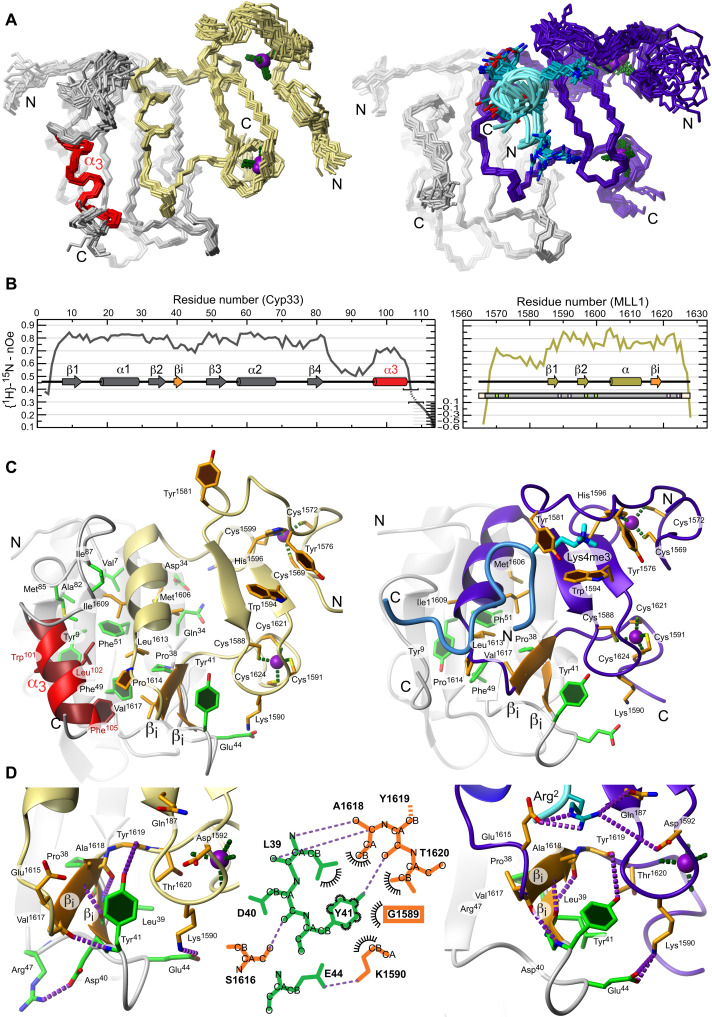
Solution structures of the RRM domain of Cyp33 bound to MLL1 PHD3 and ternary complex among Cyp33, MLL1, and H3K4me3. (**A**) Twenty energy lowest conformers in backbone representation. Left: MLL1 third plant homeodomain (PHD3) (Kaki) bound to Cyp33 RRM (gray); right: MLL1 PHD3 (purple) bound to Cyp33 RRM∆α and H3K4me3 (cyan). (**B**) Heteronuclear nOe of Cyp33 RRM bound to MLL1 PHD3 with indicated secondary structure elements. (**C**) Most representative conformers in cartoon and stick representation of the same structures as in (A). (**D**) Close-up views with schematic illustration of the loop between the second and the third β strand (β2β3-loop) region with the key residue Y41 and the intermolecular β strand complementation in binary and ternary complex.

The NMR spectrum of MLL1 PHD3 (residues 1564 to 1627; Fig. 1B) in the free state (fig. S3E) was similar to those published previously (*4*, *28*). We performed NMR titration experiments with Cyp33 RRM (fig. S3E). In contrast to the titrations of Cyp33 RRM with RNA, backbone amide resonances of this protein-protein complex were in slow exchange with respect to the NMR time scale, in agreement with previously published data (*4*). Using dihedral backbone angle restraints derived from TALOS+ (*38*) and nOe-based distance restraints, including 226 intermolecular ones (Table 1 and fig. S3F), we could determine a precise structure of the complex (Fig. 3A).

**Fig. 4.**
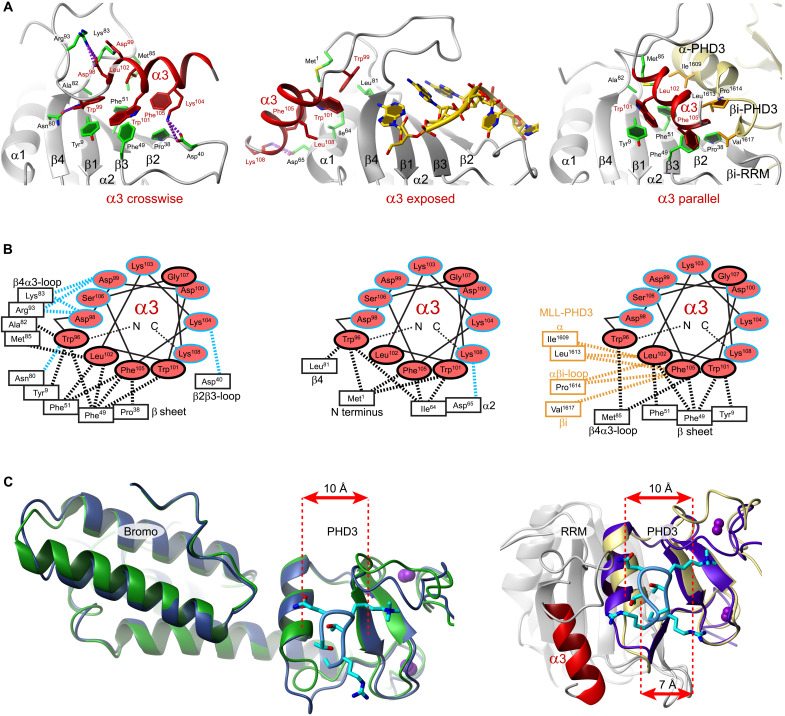
The three conformations of the α3 helix and the molecular basis for allosteric regulation of MLL1 and H3K4me3 by Cyp33. (**A**) Cartoon representations of α3 crosswise to the β sheet (ligand-free state) exposed (RNA-bound state) or parallel (MLL1-bound state). (**B**) Helical wheels with schematic interactions between α3 and the β sheet, the N-terminal region, or MLL1 PHD3 for the three conformations as in (A). Polar interactions are colored blue and nonpolar black. (**C**) α3-dependent closing of the binding cleft for H3K4me3 upon interaction with Cyp33. Left panel: Superimposed structures of MLL1 PHD3–bromodomain (BRD) free [green; Protein Data Bank (PDB) code 3LQH] and bound to H3K4me3 (blue and cyan; PDB code 3LQJ). Structures published by Wang *et al*. (*13*). Right panel: Superimposed structures of Cyp33 RRM (gray) with indicated α3 (red) bound to MLL1 PHD3 (kaki) and the ternary complex among Cyp33 RRM∆α (gray), MLL1 PHD3 (purple), and H3K4me3 (cyan).

The mobile regions of Cyp33 RRM, namely, the β2-β3 loop and the α3 helix, undergo conformational changes due to interactions with MLL1 PHD3. As confirmed by increased ^15^N-[^1^H]-nOe values compared to the free state (Fig. 3B), the Cyp33 β2-β3 loop becomes ordered upon complex formation, as it interacts extensively with MLL1 (see below). In addition, α3 is also more rigid, lying above the β strands but now in a parallel orientation (Fig. 3C). This new position differs from the two positions seen in the free protein or in the complex with RNA where α3 was perpendicular to the β strands or pointing away from the β sheet, respectively (Fig. 4, A and B). In this Cyp33 RRM/MLL1 PHD3 complex, α3 shares the hydrophobic surface of the β sheet with the α helix of the PHD3. In addition, the two helices interact via Leu^102^ in Cyp33 α3 and Ile^1609^ and Pro^1614^ in MLL1 helix (Fig. 3C).

Comparison between MLL1 PHD3 free (*28*) and bound to Cyp33 RRM shows differences at the interface, namely, the helix and the following loop, which forms a small intermolecular β sheet with Cyp33 β2-β3 loop (Fig. 3). Formation of this βi-βi intermolecular β sheet upon complex formation explains the large chemical shift perturbations seen in the PHD3 domain (Fig. 3D and fig. S3E). Backbone ^15^N-[^1^H]-nOe data of the Cyp33 RRM/MLL1 PHD3 complex shown in Fig. 3B suggest elevated dynamics for the loop region before the β-turn of MLL1 PHD3 (residues 1575 to 1585). For a more detailed analysis of the molecular interactions between Cyp33 RRM and MLL1 PHD3, see fig. S3 (G to I). Together, this protein-protein complex presents a large interaction surface involving again Cyp33 α3. This interface’s solvent-accessible surface amounts to 1267 Å^2^, which explains the micromolar affinity of the complex (4.6 μM *K*_d_; table S1) and the slow exchange regime seen by NMR during complex formation. The structure also reveals that the interface used by Cyp33 RRM to bind RNA is occluded by MLL1 PHD3, in agreement with competition assays performed previously (*4*).

### The third α helix of Cyp33 RRM regulates the binding of MLL1 PHD3 to H3K4me3

As shown above, when Cyp33 RRM is bound to RNA, it can bind to MLL1 PHD3/H3K4me3, leading to the dissociation of both the RNA and the H3 tail to form a stable complex with the PHD3. The RNA and the H3 tail thereby further shift the interaction equilibrium by stabilizing each other (fig. S3, B and C). Puzzled by the above results, we investigated the binding affinity of the stable Cyp33 RRM/MLL1 PHD3 complex for H3K4me3. ITC was used to determine the binding affinity (table S1 and fig. S2E). We measured a weak binding (*K*_d_ of 70 μM), weaker than the binding of the PHD3 domain alone (*K*_d_ of 51 μM). Because Cyp33 α3 is in interaction with MLL3 PHD3, we then wondered whether α3 might not be responsible for the weaker affinity of MLL3 PHD3 for the histone mark. We therefore measured the affinity of H3K4me3 to Cyp33 RRM∆α/MLL1 PHD3, which resulted in an unexpected higher affinity with a *K*_d_ value of 24 μM, which is three times stronger than for Cyp33 RRM/MLL1 PHD3 and two times stronger than for MLL1 PHD3. This indicated that a trimolecular complex among Cyp33, MLL1 PHD3, and H3K4me3 could be formed, in principle, but three times more favorably without α3.

We therefore determined the solution structure of MLL1 PHD3 bound to both Cyp33 RRM∆α and H3K4me3 (Fig. 3, A, C, and D, right side of the panels). To a large extent, the binding of H3K4me3 kept intact the interface between MLL1 PHD3 and its second binding partner Cyp33 RRM, despite a slightly different binding position of the α helix of PHD3 relative to the RRM β sheet (Fig. 3C). Furthermore, interactions with H3K4me3 including the aromatic cage formed around the methylation marks were very similar to those observed in the complex of H3K4me3 bound to the PHD3-BRD fragment of MLL1 (*13*). Despite the close proximity of Cyp33 RRMΔα β2β3-loop and H3K4me3, they do not interact.

**Fig. 5.**
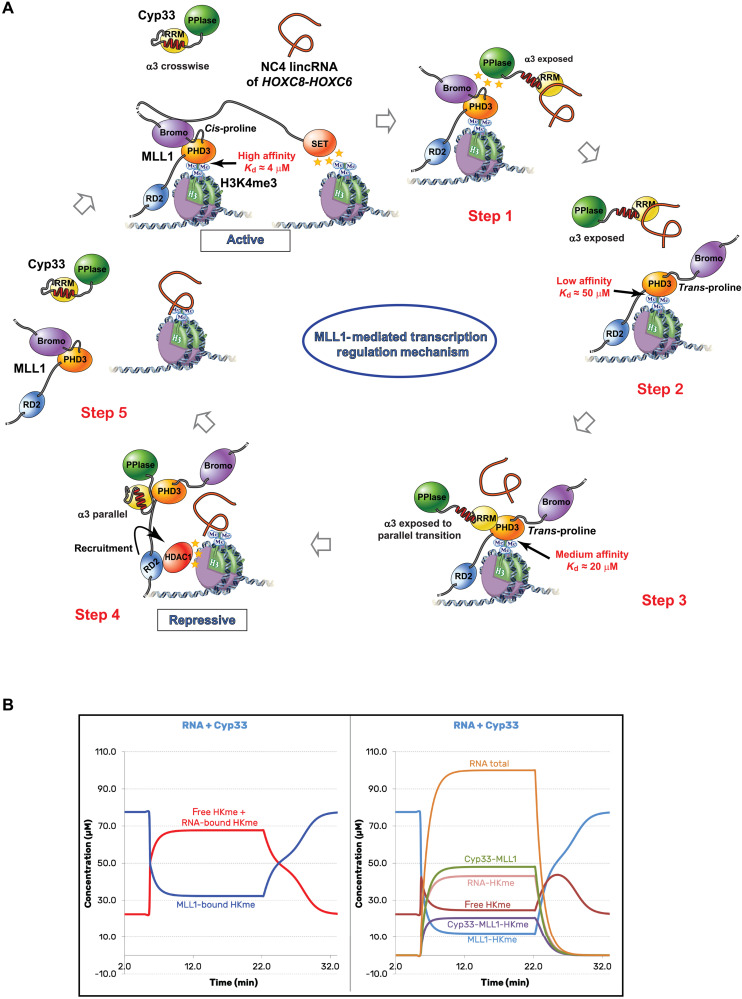
The third α helix of Cyp33 RRM causes an RNA-dependent allosteric regulation of MLL1 PHD3 binding to epigenetically marked Histone H3. (**A**) MLL1 promotes the transcription of long intergenic noncoding RNAs (lincRNAs) *NC3/NC4*, which are bound by Cyp33 RRM when the expression level of these RNAs increases (step 1). This interaction induces a change in position of Cyp33 a3 helix that allows its interaction with MLL1 (steps 2 and 3), forces the release of the protein from H3K4me3, and represses transcription by a negative feedback (step 4). The interaction of RNA with H3K4me3 helps to release the histone tail from MLL1 (step 5). (**B**) Equilibrium populations of different species in the target system, as simulated by the developed ODE model encompassing all underlying reactions. At *t* = 0 min, the initial system is equilibrated with only MLL1 and H3K4me at 100 μM each. At *t* = 5.6 min, RNA transcription is triggered, simultaneously recruiting Cyp33 to the system—with each species reaching 100 μM concentration at its maximum—thus giving equimolar concentrations for all four components of the system (MLL1, H3K4me, RNA, and Cyp33). At *t* = 22 min, RNA transcription is turned off, allowing gradual removal of RNA and Cyp33 from the system. Left panel shows the balance between the “active” (blue trace) and repressive (red trace) states of H3K4me. The active state combines the binary MLL1-H3K4me and tertiary Cyp33-MLL1-H3K4me complexes. The repressive state combines the free H3K4me and RNA-bound H3K4me. Right panel shows the corresponding time-resolved dynamics for each of the above species separately.

This absence of contacts between Cyp33 and H3K4me3 suggests that Cyp33 uses its RRM α3 to allosterically regulate MLL1 binding to H3K4me3. By comparing the PHD3 binding pocket for H3K4me3 in our solution structure of Cyp33 RRM∆α/MLL1 PHD3/H3K4me3 with the crystal structures of the free MLL1 PHD3-BRD [Protein Data Bank (PDB) 3LQH] and H3K4me3 bound to MLL1 PHD3-BRD (PDB 3LQJ) (*13*), we see in all these three structures a binding pocket for H3K4me3 which is 10 Å wide (Fig. 4C). In the free MLL1 PHD3-BRD protein, we see that this 10-Å wide cleft is already preformed due to the interactions between the BRD domain and the PHD3 (Fig. 4C). If the proline 1629 is in a *trans* conformation, then PHD3 is no longer stabilized by the BRD domain, which results in a narrowing of the cleft from 10 to 9 Å (MLL1 PHD3) (*28*). Binding of Cyp33 RRM∆α to MLL1 PHD3 seems to restabilize this 10-Å conformation. However, in the presence of α3 (Cyp33 RRM/MLL1 PHD3 complex), the two helices (α3 of Cyp33 and α helix of PHD3) compete for the hydrophobic patch present on the RRM β sheet. This, in turn, results in a squeezing of the binding cleft down to 7 Å, explaining why Cyp33 RRM/MLL1 PHD3 binds weakly to H3K4me3 and even releases H3K4me3 if Cyp33 is prebound to RNA (fig. S3). In conclusion, Cyp33 α3 appears to be a switchable element capable of regulating not only Cyp33 RNA binding but also MLL1 binding to H3K4me3. With this ensemble of new structures and the previous findings (*13*), we can now rationalize how RNA binding triggers Cyp33 binding to MLL1 and releases it from the histone tail for ultimately repressing transcription (Fig. 5A).

### Network model suggests that the switch to repressive state requires both Cyp33 and RNA

To more comprehensively understand the ensemble of interactions, we integrated our data into a network of biochemical reactions to calculate a dynamic model based on ordinary differential equations (fig. S4). The model was parameterized on the basis of the measured reaction constants from this work and data from previous literature, with additional kinetic parameters approximated based on diffusion limits (table S2). Simulations of the model indicated that the expression of both RNA and Cyp33 is needed to switch the chromatin to a “repressive” state. This is indicated by an increase of free and RNA-bound H3K4me3 tail (in red) and a strong decrease (in blue) of MLL1-PHD3 bound to the H3 tail (Fig. 5B). Presence of RNA or Cyp33 alone is insufficient to cause such rebalancing (fig. S4). This rebalancing upon simultaneous presence of Cyp33 and RNA occurs even with a conservative modeling assumption that RNA contains only a single H3K4me3 binding site. Furthermore, the slight dominance of the repressive state of the system (H3K4me3 tail not bound to MLL1-PHD3) remains even if RNA-H3K4me3 affinity is assumed very weak (500 μM) (fig. S4F). The last two points suggest that the switch of this system to the repressive state can robustly tolerate certain changes in key reaction constants of the target network in the cell nucleus environment.

## DISCUSSION

### A conserved α helix downstream of an RRM adopts three positions

In term of structural plasticity, the three positions adopted by the conserved α helix downstream of the RRM in free Cyp33, bound to RNA, and bound to MLL1 PHD3 is unprecedented (Fig. 4A). Change of position upon RNA binding of a C-terminal helix or folding upon RNA binding has previously been reported for U1A N-terminal RRM and in Polypyrimidine Tract Binding Protein 1 (PTBP1) RRM1, respectively (*39*, *40*). In both cases, the RNA induced repositioning or folding of the C-terminal helix is functionally important, as it allows U1A dimerization and stem-loop recognition, respectively (*39*, *40*). Here, the three-helix positions found in Cyp33 allow interactions with both RNA and a protein, resulting in a cascade of binding events (Fig. 5). RNA binding to Cyp33 triggers a first switch of the helix that facilitates binding to MLL1 PHD3-H3K4me3. Then, a second positional switch triggers the release of both the RNA and the histone mark from the MLL1-Cyp33 complex. A third switch leads to the dissociation of the proteins. Overall, Cyp33 senses the presence of RNA and transduces this signal toward a chromatin structure change (Fig. 5).

### Cyp33 RRM senses RNA and transduces the signal to the chromatin via MLL1 PHD3

Although the structure of MLL1 PHD3 and Cyp33 had been solved and their interaction had been studied (*4*, *13*, *28*), how Cyp33 binding to MLL1 leads to transcription repression remained a mystery, and contradictory mechanistic models have emerged. Notably, past structural works studied the RRM in isolation ignoring the evolutionary conserved C-terminal region of Cyp33 RRM. Our structural work revealed that this conserved region folds into an α helix that is critical to the function and the mechanism of action of Cyp33. By combining previous structural work with the five structures presented here, we can now propose a full mechanistic path explaining how Cyp33 when stimulated by RNA binding could change the chromatin from a transcriptionally active state to a repressive state (Fig. 5).

In the initial transcriptionally active state, MLL1 is bound near the transcription start sites of *HOX* genes. MLL1 binds H3K4me3 via its PHD3 domain and maintains a high level of this modification via its catalytic SET domain (Fig. 5). In the active state, the MLL1 PHD3 and BRD domains interact and are tightly bound to H3K4me3 (*K*_d_ of 4 μM) (*13*). Several lincRNAs are expressed in the vicinity of the *HOX* genes and regulate their expression (*41*). Among those, we show that *NC3* and *NC4* are bound by Cyp33 sequence specifically (Fig. 2) probably due to the presence of multiple copies of the YAAUNY RNA binding consensus sequence, which is an optimal binding sequence for the RRM of Cyp33. Although the binding affinity of Cyp33 for a single RNA motif is weak (*K*_d_ of 300 μM; table S1), the affinity is increased by avidity due to the presence of multiple copies of this motif. The lincRNAs *NC3* and *NC4* could therefore recruit Cyp33 to the site of transcription of the *HOX* gene and in proximity to MLL1 using a very sophisticated mode of regulation. The interaction of the α3 helix with the RNA binding interface of Cyp33 in its free form prevents a premature recruitment of the protein at the transcription site. A minimal amount of transcribed lincRNA will be needed to compete out the α3 helix from the β sheet surface and initiate the repressive mode of action of Cyp33. The Cyp33-MLL1 interaction happens then in two steps. First, Cyp33 PPIase binds to MLL1 and induces the isomerization of the Pro^1629^ from *cis* to *trans*, which weakens the interaction between the BRD domain and the PHD3 but still maintains the H3K4me3 bound to the PHD domain (Fig. 5A, steps 1 and 2) (*13*). The interaction with RNA also translocates the Cyp33 α3 helix on the side of the RRM (toward β4; Fig. 4A, middle), preparing the β sheet surface for subsequent interaction with MLL1 PHD3. Now, both the PHD3 domain and Cyp33 RRM are in a conformation that is optimal for them to interact. Although RNA is bound to the Cyp33 RRM, the affinity of PHD3 for the RRM is much stronger (60-fold, i.e., *K*_d_ of 5 versus 300 μM). So contrary to what was anticipated, RNA binding does not inhibit Cyp33-MLL1 interaction but would rather stimulate it by recruiting Cyp33 to the site of transcription and by repositioning the α3 helix to facilitate its binding to MLL1. Our data suggest that the complex in step 3 is only transient (Fig. 5). The dissociation constant of H3K4me3 from the MLL1/Cyp33 RRM complex is higher (*K*_d_ of 70 μM) than for MLL1/Cyp33 RRMΔα3 (*K*_d_ of 24 μM) due to the interaction of Cyp33 α3 with the α helix of MLL1 PHD3 (Fig. 3C). Therefore, the interaction of Cyp33 α3 with the MLL1 PHD3 results in a squeezing of the histone binding pocket and dissociation of H3K4me3 (Fig. 5A, step 4). We proved this step experimentally when mixing at a stoichiometric ratio Cyp33/RNA with MLL1 PHD3/H3K4me3, as it resulted in the formation of a Cyp33/MLL1 PHD3 complex and the release of both the RNA and H3K4me3 (fig. S3). The fact that the RNA and H3K4me3 interact, further pushes the equilibrium toward almost full dissociation of the histone mark from MLL1 (Fig. 5). This interaction between the RNA and the histone tail is further supported by recent publications, indicating that the nucleosome histone tails, and in particular H3K4, do interact with RNA (*42*, *43*). The H3K4me3 mark is now accessible to histone demethylases and histone deacetylases, ultimately leading to a repressive transcriptional state of the chromatin (Fig. 5, step 4). With the decrease in RNA concentration, Cyp33 and MLL1 should ultimately dissociate via their intramolecular interactions (between α3 and the RRM in Cyp33 and between the PHD3 and the BRD domains in MLL1).

In summary, the proposed mechanistic path derived from our structural and biochemical work now explains why Cyp33 can repress MLL1-derived transcription and how this is triggered by RNA binding of Cyp33 (most probably lincRNAs). Our results reveal a very sophisticated mechanism of negative feedback regulation of transcription mediated by Cyp33, RNA, and MLL1 (Fig. 5).

### MLL1-Cyp33, a cooperation that leads to lincRNA-mediated transcription regulation

We propose here that MLL1 could promote the transcription of lincRNAs *NC3* and *NC4*, which are bound by Cyp33 RRM when the expression level of these RNAs increases. This interaction induces a change in position of Cyp33 α3-helix that allows its interaction with MLL1, forces the release of the protein from H3K4me3 and represses transcription by negative feedback (Fig. 5). The involvement of MLL1 in inducing transcription of lincRNAs is not an isolated case. MLL1 has a key role in inducing transcription of the lincRNA *HOTAIR* under hypoxia in several types of cancer cells (*44*). Previous studies reported interactions of MLL1 associated to other proteins with lincRNAs. For example, *Fendrr* lincRNA can interact with the TrxG/MLL complex and was shown to form a double-stranded DNA/RNA triplex, allowing the recruitment of the polycomb repressive complex 2 and subsequent H3K27 trimethylation, a repressive histone mark, at specific target sites (*45*). Conversely, the lincRNA HoxBlinc was shown to recruit the Setd1a/MLL1 complex to activate transcription of *HoxB* genes (*46*). In addition, it was shown that a chromosomal looping could bring the WDR5/MLL complex across the *HOXA* gene to promote gene transcription (*41*). On the basis of a protein mutant that affects the interaction of WDR5 with RNA but not with the MLL complex, it was proposed that lincRNAs could bind to WDR5 to stabilize its interaction on chromatin, which facilitates the subsequent assembly of the MLL complex and gene activation (*47*). All these data suggest that lincRNAs can regulate gene expression by attracting positive or negative epigenetic regulators to specific chromatin sites bound by MLL1. These other regulatory pathways use other parts of MLL1 than the PHD3 domain. Although the mode of binding of these large regulatory complexes bound to a single nucleosome were recently solved by cryo–electron microscopy (*48*, *49*), the mode of action of RNA in these regulatory processes remain elusive. Last, the mode of action of Cyp33 in transcription repression resembles the mode of action of RNA Binding Fox-1 (RBFOX-1) (*50*) and of the RNA-induced silencing complex (RISC) in transcription regulation (*51*) except that Cyp33 recruitment to chromatin results in the release of MLL1, while both RBFOX-1 and RISC lead to the recruitment of a repressor complex.

In summary, we structurally and functionally addressed open questions on the mechanism of Cyp33 regulated and MLL1-mediated gene expression. Initially, it was not clear how RNA binding results in a cross-talk between the two domains of Cyp33. Furthermore, a major contradiction between two models built on the existence or not of a ternary complex among Cyp33, MLL1, and the histone H3 was existing in literature. Namely, whether MLL1 remains bound to H3K4me3 in its repressive state and Cyp33 provokes through an unknown mechanism the recruitment of co-repressors (*13*) or whether binding of Cyp33 to MLL1 results in histone H3 dissociation with a concomitant repression (*28*). Our results revealed that the RRM domain of Cyp33 has a C-terminal third α helix that plays a central role in the regulation of MLL1-mediated gene expression by Cyp33. In addition to be the molecular sensor of RNA binding to the RRM, α3 helix allosterically dictates Cyp33 interaction with MLL1 and forces the protein to be released from the specific activation marks in the histone H3 leaving them exposed for epigenetic erasers. The enigmatic role of RNA in this process seems to play a more critical function than initially anticipated. Our data indicate that RNA could potentially not only recruit Cyp33 but also help to release MLL1 from H3K4me3 by interacting with the histone tails. It opens unexpected perspectives on RNA-mediated gene regulation, which can now be investigated further in cells. Leukemogenic MLL1 variants lack the entire homeobox and all adjacent domains including the writer domain SET (*22*). In agreement with our conclusions, it was shown that the sole reinsertion of PHD3 restores Cyp33 recruitment and rescues the aberrant transcription caused by MLL1 oncogenic fusion proteins (*26*, *27*). Hence, its involvement in this interaction network makes Cyp33 a key player for the understanding of the oncogenic nature of MLL1 in infant leukemia in particular and potentially the mechanism of leukemogenesis in general.

## MATERIALS AND METHODS

### Protein expression and purification

Vectors encoding the Cyp33 RRM∆α and Cyp33 RRM constructs were transformed into chemical competent BL21-CodonPlus (RIL), and the vector encoding the codon-optimized construct for MLL1 PHD3 was transformed into chemical competent BL21-(DE3) *Escherichia coli* cells. All proteins were expressed using the IMPACT (Intein-Mediated Purification with an Affinity Chitin-binding Tag) expression system. Expression was performed in either LB for unlabeled protein or M9 minimal medium enriched with ^13^C-glucose and/or ^15^NH_4_Cl for ^15^N and ^13^C or only ^15^N labeling schemes.

The cells were grown at 37°C until the optical density at 600 nm (OD_600_) reached 0.8 and were then induced with 0.5 mM isopropyl-β-d-thiogalactopyranoside at 20°C and incubated for another 24 hours. The cells were harvested, centrifuged, and resuspended in 30 ml of lysis buffer [30 mM Hepes and 0.5 M NaCl (pH 8.0)] and 3 μl of 1 M phenylmethylsulfonyl fluoride protease inhibitor. This cell suspensions were lysed using a M110S homogenizer of Microfluidics and purified on chitin beads (New England Biolabs) by washing with lysis buffer, high salt buffer [30 mM Hepes and 2 M NaCl (pH 8.0)], and again lysis buffer. In case of purification of one of the Cyp33 variants, intein autocleavage was induced with lysis buffer containing 50 mM dithiothreitol (DTT) followed by at least 12 hours of incubation at room temperature and subsequent elution using twice 20 ml of NMR buffer [40 mM KCl and 20 mM KH_2_PO_4_ (pH 7.0)]. Cleavage of MLL1 PHD3 protein could not be achieved by intein autocleavage, because DTT at high concentrations has the tendency to complex Zn^2+^ ions. Instead, 1 mg of sequence specific protease from Tobacco Etch Virus (TEV) protease was added onto the chitin column and incubated overnight at room temperature. Except the addition of 10 μM ZnCl to the NMR buffer, the same elution protocol was applied as described for the Cyp33 variants. The NMR buffer was selected using differential scanning fluorimetry. In this method, the melting temperature of a protein is tested in presence of a variety of buffer conditions (96 conditions) and a dye with affinity for the hydrophobic parts of the protein by monitoring fluorescence-based thermal shifts (*52*). The conditions that were selected in the end are both keeping the protein stable and suitable for solution NMR experiments.

The elution products were concentrated to a volume of less than 1 ml using Vivaspin 2 centrifugal concentrators with a 10-kDa cutoff for the Cyp33 variants or a 5-kDa cutoff for the MLL1 PHD3 protein and further purified by size exclusion chromatography using a Superdex 75 10/300-GL column in according NMR buffer. For Cyp33 variants to be used for addition of RNA, 10 μl of SUPERase•In Ribonuclease Inhibitor (Ambion) were supplemented to the sample before applying it to the column.

### NMR spectroscopy, structure calculation, and refinement

NMR spectra were acquired at 303.15 K for the free Cyp33 RRM domain and at 310.15 K for all complexes. Triple-resonance experiments [experiment correlating atom names HN and CA (HNCA) and CBCAcoNH] for backbone assignment and three-dimensional (3D) Total Correlation SpectroscopY (TOCSY) experiments (hCccoNH and HcccoNH) for side-chain assignments (*53*) were collected at 500, 600, or 700 MHz using Bruker Avance III spectrometers equipped with TCI cryoprobes. Spectra dedicated for RNA resonance and nOe assignment were collected using samples in 100% D_2_O and 90% H_2_O/10% D_2_O for all other purposes. Homonuclear 2D, ^15^N- and ^13^C-edited 3D Nuclear Overhauser and Exchange Spectroscopy (NOESY) experiments for structure calculation and assignments of aromatic residues were all acquired on a Bruker Avance III HD 900 spectrometer equipped with a TCI cryo probe (*T*_M_ = 120 ms). All spectra were processed with TopSpin 3.0 and analyzed with Sparky 3.1.1.4.

RNA resonance assignment was achieved using [^1^H-^13^C]-HSQCs (Heteronuclear Single Quantum Coherence) using the natural abundance of the ^13^C isotope, homonuclear 2D TOCSY (spin_lock = 50 ms), 2D NOESY (*T*_M_ = 120 ms), and ω2-filtered 2D NOESY (sample with ^13^C-labeled protein). Assignments of nOes were based on the manual analysis of ω3-filtered ^13^C-resolved 3D NOESY (*54*) and homonuclear 2D NOESY experiments for the protein RNA complex with ^13^C-labeled protein and based on automated analysis (as described in next section) of standard ^15^N- or ^13^C-resolved 3D NOESY experiments for the protein-protein complex with one, the other, or both protein components, ^13^C and ^15^N labeled.

Backbone ^15^N-[^1^H] heteronuclear nOes were measured on a Bruker Avance III 750 spectrometer equipped with a TCI room temperature probe at a transmitter frequency of 750.134 MHz for the proton and 76.019 MHz for 15 N (*55*). For the backbone ^15^N-[1H]-nOe and for the reference experiment, a relaxation delay of 2 s and a water gate solvent suppression was used.

Regarding the structure calculation, initial peak picking and nOe assignments was performed using the ATNOS/CANDID package. For NOESYs of nonuniformly labeled samples, e.g., only one component was labeled, ATNOS/CANDID had to be aborted after completing the first cycle, and the consolidated shift list concatenated to the according NOESY had to be modified. Hence, if ATNOS/CANDID performed the peak picking of an ^13^C-resolved 3D NOESY where only MLL1 PHD3 was ^13^C labeled but not Cyp33-RRM, then shift assignments of all given shift lists were consolidated. Thus, the respective shift list contains ^13^C shifts of MLL1 PHD3 and Cyp33 RRM. This led to the problem that peaks were potentially interpreted wrong, caused by picked artifacts. To overcome this problem, the according shift list in cycle one was manually modified by deleting all resonance assignments, which were not detected by manual inspection of the respective NOESY experiment, and ATNOS/CANDID was restarted from cycle two. Because RNA cannot be interpreted by ATNOSCANDID standard library, only protein shifts were given, and nOe signals of RNA were manually picked, assigned, calibrated, and further used as distance restraints.

Peak lists of the final seventh cycle and manual derived restraints involving RNA were used as an input for the program CYANA 3.0 (*56*). The “noeassign” protocol of CYANA was used to reassign and calibrate the nOe signals of the given peak lists, resulting in protein-protein restraint lists. These lists were cleaned by applying a cutoff for the quality factor of 0.5 and by reviewing the peak lists and inspection of the NOESY spectra. Including all distance restraint lists and, in some cases, torsion angle restraints for the protein backbone derived by TALOS+ (*38*) or sugar pucker torsion angle restraints of RNA based on coupling efficiency in the homonuclear 2D TOCSY, CYANA was further used to calculated 250 structures by a simulated annealing protocol (MD, molecular dynamics steps = 20,000). On the basis of the target function, the 50 best structures were selected for refinement.

The AMBER 9 package (*57*) was used for structure refinement in the presence of the force field ff99SB (*58*) and implicit solvent [generalized Born model to mimic water as described in (*59*)]. Harmonic square-well penalty functions with force constants of 20 kcal mol^−1^ Å^−2^ for distance restraints and 300 kcal mol^−1^ rad^−2^ for torsion angle constraints were applied. First, a short minimization with long-range electrostatics treatment by the particle mesh Ewald method (*60*) using steepest descents energy minimization, followed up with conjugate gradient minimization was performed. The minimized structures were then refined using a simulated annealing protocol of 30,000 steps. For all refinements, 1-fs time steps in combination with constraint bond lengths by applying SHAKE (*61*) and 15-Å nonbonded cutoff were used. Scaling factor for the one to four electrostatic and one to four nonbonded van der Waals interactions were set to default values as used for the parameterization of the ff99SB force field (scee = 1.2 and scnb = 2.0). The details of applied input temperature, restraint ramping, and actual system temperature over the course of refinement can be seen in fig. S4. From the 50 structures refined in AMBER, 30 structures with the lowest AMBER energy were preselected, from which 20 structures with the lowest violation energy were selected for the final representative ensemble. The statistics for these ensembles can be seen in Table 1.

## Supplementary Materials

### This PDF file includes:

Supplementary Materials and Methods Figs. S1 to S4 Tables S1 and S2 References
