# Supplementary material for: RNA binding induces an allosteric switch in Cyp33 to repress MLL1-mediated transcription
Source: Sci Adv. 2023 Apr 19;9(16):eadf5330. doi: 10.1126/sciadv.adf5330 (PMC10115415; doi:10.1126/sciadv.adf5330)
Supplement: Supplementary file 1 — Supplementary Materials and Methods Figs. S1 to S4 Tables S1 and S2 References [file sciadv.adf5330_sm.pdf]

Science Advances
AAAS

Supplementary Materials for

RNA binding induces an allosteric switch in Cyp33 to repress MLL1-mediated transcription

Markus Blatter *et al.*

Corresponding author: Frédéric H.-T. Allain, allain@bc.biol.ethz.ch; Markus Blatter, blatter.markus@novartis.com

Sci. Adv. **9**, eadf5330 (2023)
DOI: 10.1126/sciadv.adf5330

This PDF file includes:

Supplementary Materials and Methods
Figs. S1 to S4
Tables S1 and S2
References

SUPPLEMENTARY MATERIALS AND METHODS

Cloning and mutagenesis

DNA encoding the RNA recognition motif domain of human Cyp33 (residues 1-90; Cyp33 RRM$\Delta\alpha$, UniProtKB entry Q9UNP9) and DNA encoding the RRM plus an additional 24 conserved amino acids (residues 1-114; Cyp33 RRM) were isolated by PCR amplification from cDNA obtained from total RNA extraction of HEK293 cells. DNAs encoding Cyp33 RRM$\Delta\alpha$ or Cyp33 RRM were subcloned into pTYB12 expression vector (New England Biolabs) using NdeI plus XhoI restriction sites and standard cloning procedures (*63*) yielding a cleaved expression product with an additional three amino acids (Ala,Gly,His) N-terminal of the target protein.

DNA encoding a short fragment of the TEV protease cleavage site and the longer fragment encoding the PHD3 of MLL1 (residue 1564- 1627; MLL1 PHD3, UniProtKB entry Q03164) was derived by a two-step protocol PCR amplification using four partially overlapping primers which were designed to be codon optimized for E. Coli wild strain. The resulting DNA encoding MLL1 PHD3 was subcloned into a pTYB11 expression vector (New England Biolabs) using standard cloning procedures (*63*) yielding a cleaved expression product with an additional serine N-terminal of the target protein.

SELEX

The SELEX experiment was performed essentially as described in Sakashita et al. (*64*).

Cloning: The full-length CYP33 coding sequence was cloned into the EcoRI and NotI sites of the pGEX-4T-3 vector (Amersham Biosciences) downstream of the GST coding sequence. The coding sequence for the CYP33 spacer and cyclophilin domain, without the RRM domain (CYP33□RRM) cloned into the EcoRI and NotI sites of the pGEX-4T.

Protein expression: Protein expression was performed using transformed BL-21 competent cells (Stratagene) and the supplier's protocol for GST tagged protein. One litre of the transformed culture in exponential growth phase was generated as per protocol and the protein expression was induced using 1mM IPTG (Isopropyl β-D-1 thiogalactopyranoside from Sigma) and incubation for 4 hours at room temperature under aeration conditions. Lysis, protein binding to separation beads and washing was done using appropriate buffers as described below: Lysis Buffer- Saline Tris EDTA buffer (10mM Tris at pH 8.0, 150mM NaCl containing 100µg/ml of lysozyme)5mM DTT (Sigma), 1X protease inhibitor cocktail tablet (Roche) 1.5% N-laurylsarcosine, and 2% Triton X with intermittent sonication and/or French Press. The lysate was centrifuged at 10,000 rpm/30min/4°C and the supernatant incubated with 1ml STE washed Glutathione Sepharose 4B (GE Healthcare) overnight at 4°C. The protein bound beads were washed with Washing buffer (20mM Tris pH8.0, 120mM NaCl, 10% Glycerol, 0.1% Triton X and 1X mini protease inhibitor cocktail). Finally, the bound protein was eluted using Elution Buffer (10mM Tris pH 8.0, 50mM reduced glutathione and 10% glycerol). Further purification was performed using Size Exclusion filter columns from Millipore following manufacturer's protocol.

RNA library for selection by ligand binding: A random RNA pool was generated using a dsDNA template with a T7 polymerase promoter site followed by 30 nucleotides long randomized sequence and a 3' constant primer binding site. The following primers were used to design the dsDNA template: T7Univprimer2

: 5'CATCTGCAAGTACTAGAGTAATACGACTCACTATA*G*GACTGACCTAGTCTGAC 3', Biotin-RevUnivPrimer2: 5' Bio/CTGACACTGCAGTCTGAG 3', LinearN30: 5' CTGACACTGCAGTCTGAG**(N$_{30}$)**GTCAGACTAGGTCAGTC 3', Biotin SlxHeel: 5' Bio/CATCTGCAAGTACTAGAG. Preparation of the ds-transcription template and in vitro transcription were performed using Ambion's MegaShortScript kit and the RNA thus obtained was cleared of dsDNA template using Streptavidin agarose following which, the RNA was pre-cleared using Glutathione Sepharose. The random RNA thus generated was bound to 2 µg or 60 nmoles of Glutathione Sepharose 4B bound GST-CYP33 in a binding buffer (200 mM HEPES-NaOH pH 7.9, 200mM KCl, 5% Glycerol, 1X protease inhibitor cocktail from Roche) at 4°C for one hour. The bound protein-RNA complex was separated from the reaction, washed and eluted for RNA purification. 20% of this recovered RNA was reverse transcribed into the first strand cDNA as described in the first step. PCR was used to generate the dsDNA template for the second round of in vitro transcription and selection. After three rounds of binding, the dsDNA thus generated was T/A cloned into the pGEMT-Easy cloning vector (Promega) and sequenced. Fifty DNA sequences representing bound RNA were screened for enriched motives. Chi square distribution tests were used to determine if the identified motives were present at frequencies significantly different from their expected random occurrence, within sequences of the average base composition of the selected sequence collections.

Preparation of RNA, peptide and complexes

Oligoribonucleotides were purchased from Dharmacon Inc. (USA), deprotected according the vendors instructions and resuspended in D_2O (2 µmol / 100 µl). The peptide composed of the first 13 amino acids of the Histone H3 N-terminal tail and tri-methylated at the ammonium of lysine four (H3(1-13)K4me3) was purchased from ChinaPeptides CO. LTD in >98% purity and resuspended in H_2O (5 mg / 100 µl). Concentrations were measured using the extinction coefficient at 260 nm for RNA (ε_{260} provided by Dharmacon), at 280 nm for Cyp33 variants and MLL1 PHD3 (ε_{280} calculated using the program DNAman) and at 205 nm or 215 nm plus 220 nm (absorption of the peptide bond) for the H3(1-13)K4me3 peptide. For Cyp33 variants complexed with MLL1 PHD3 a mixture of 1:1.1 was repurified by size exclusion chromatography yielding an exact stoichiometry of 1 to 1. Protein-RNA complexes or ternary complexes of Cyp33 RRM – MLL1 PHD3 with H3(1-13)K4me3 were either titrated (monitored by a series of [1H-15N]-HSQCs) or mixed to desired stoichiometry (usually slight excess of RNA/peptide) according the measured concentrations. Final sample concentrations ranged from 1mM to 2mM. Samples in D_2O were prepared by lyophilization and resuspension in D_2O.

NMR titrations

Multi-complex assembly/dissociation: H3K4me3 peptide was titrated at 310°K with MLL1 PHD3 in 20mM $KHPO_4$ pH7, 40mM KCl, 50µM $ZnCl_2$ buffer to a 1:1 ratio. Cyp33 RRM was then titrated with UAAUGU RNA to a 1:1 ratio in the same conditions. Finally, both complexes were mixed and NMR signals were followed upon time. The first serial file of F1-filtered 2D NOESY was used to follow the evolution of unlabelled RNA and K4me3 group of unlabelled peptide and ^{1}H-^{15}N HSQC to follow the evolution of ^{15}N-labeled protein amides.

Affinity determination: NMR titrations of UAAUGU RNA with Cyp33 RRM WT or mutants were carried out at 289.15K in 20mM $KHPO_4$ pH7, 40mM KCl, 1mM DTT buffer to a ratio of up to 6:1 and chemical shift changes were followed in a series of ^{15}N-^{1}H-HSQC experiments.

All titration experiments were measured on a Bruker AVII-700MHz spectrometer equipped with a cryoprobe.

Reaction network model

The model based on ordinary differential equations was formulated with rule-based modeling in BioNetGen language (*65*). Model simulations in different conditions were performed using RuleBender package (*66*). Model parameters are listed in table S2. The final model was deposited in BioModels (MODEL2201310002).

Isothermal titration calorimetry (ITC)

In order to prepare the receptor and ligand molecules or complexes, each macromolecular component was dialyzed against the NMR buffer and concentrated to a stock solution. The flow through was kept separately. Concentrations of proteins and RNAs were determined using optical density absorbance at 280 and 260 nm, respectively. Components for the calorimetric cell were prepared by diluting the protein or protein complexes with the flow through (NMR buffer). For the experiments using the H3K4Me3 peptide as ligand the receptor concentration was adjusted to 50 µM concentration and for all other experiments 20 µM receptor concentration. The ligand concentration for the syringe varied between 400 µM and 600 µM except for the peptide, which was prepared at 2 mM concentration. For the sole ITC experiment with RNA, a 500 µM solution of the Cyp33 RRM WLF protein was filled into the syringe and 20 µM of UAAUGU RNA into the cell. For all other experiments component in the syringe was either MLL1 PHD3 or H3K4me3. Finally, the data shown in Figure S2F was performed by titrating 1mM of H3K4Me3 (in the syringe) into a 10µM RNA solution.

In order to have more measuring points at the start of the sigmoidal curve, for some of the experiments the initial 10 injections volumes were set to 4 µl followed by injections of the normal 8 µl, leading to the

apparent discontinuity in the decrease of the heat response amplitude. Total number of injections were set to 40 with a delay of 5 minutes and measurement stopped automatically after syringe volume was consumed. All experiments were performed at 25°C on a VP-ITC instrument (Microcal), calibrated according to the manufacturer's instructions. Raw data were integrated, normalized for the molar concentration, and analyzed using the Origin 7.0383 software according to a one binding site model.

PPIase activity assay

The PPIase activity of recombinant Cyp33 wt was performed as described (*67*) by using the tetrapeptide substrate Suc-AAPF-pNA (N-succinyl-Ala-Ala-Pro-Phe p-nitroanilide; Sigma-Aldrich). All reagents were pre-equilibrated until the temperature reached 4°C. In a 1-ml glass cuvette, 5-40 nM Cyp33-wt was mixed with 100 µl of α-chymotrypsin (Sigma-Aldrich; 60 mg/ml in 1 mM HCl), and the volume was adjusted to 975 µl with assay buffer (50 mM Hepes-KOH, pH 8.0 at 0°C, 100 mM NaCl, 2 mM $MgCl_2$, 1 mM EDTA). The reaction was initiated by the addition of 25 µl of substrate (4 mM tetrapeptide in 470 mM anhydrous LiCl dissolved in trifluoroethanol). Changes in absorbance due to released p-nitroaniline were monitored at 390 nm at 4°C over a 2-min period in a Carry 300 BIO (VARIAN) UV/VIS spectrophotometer with a thermostatically controlled cuvette holder. Condensation was avoided by a cooled airflow using a BEKODRYPOINT-M (BEKO). To check PPIase activity of proteins in presence of RNA, samples with all five protein concentrations were pre-incubated in reaction buffer with 200NM of 7 nt RNA (UAAUGU). All experiments for Cyp33-wt free and bound to RNA were performed three times with different preparations of proteins. The catalytic PPIase activity was calculated from the slope of the K_{obs} vs. protein concentration.

RT-PCR

HEK293T cells were transfected with pCEP4 plasmid constructs expressing full length FLAG-tagged wild-type and mutated versions of CYP33 using Lipofect-amine 2000 (Invitrogen). After 48h, RNA was harvested using Trizol (Invitrogen) and reverse-transcribed. Quantitative PCR was performed in triplicate using GoTaq qPCR Master mix (Promega) on an CFX96 Real-Time PCR (Biorad). Expression levels of *HOXC8, HOXC9* and *Six1* were normalized to *β2M.*

UV-crosslinking RNA immunoprecipitation (UV-RIP)

HEK293 cells were transfected with pCEP4 plasmid constructs expressing full length FLAG-tagged wild-type and mutated versions of CYP33. UV-RIP protocol was taken and modified from Jeon and Lee, and Cabianca et al. (*68, 69*). Briefly, cells were harvested 24h after transfection, washed 1x with PBS1x and UV-crosslinked on ice with 2x irradiations of 100000 µJ/cm^2. Afterwards, cells were lysed with lysis buffer [0.5%NP-40, 0.5%NaDeoxycholate, 1xRoche protease inhibitor, 25U/mL Superase-RNAse inhibitor

(ThermoFisher Scientific) and PBS as solvent], scraped off the plate and transferred to a clean Eppendorf tube. Lysates were then rocked on a wheel for 30min at 4°C and then treated with 30U of Turbo DNAseI (ThermoFisher Scientific) for 30min at 37°C in a Thermomixer at 1100rpm. DNA-digested lysates were subsequently centrifuged for 5min at 1350g to remove debris and the supernatant was used for the immunopurification analysis. An aliquot of the supernatant (10%) was kept as input material. The remaining supernatant was split in two halves in fresh Eppendorf tubes and control IgG (Jackson Laboratory) or FLAG (Thermo Fisher Scientific) rabbit antibodies were added to each fraction, respectively. The final volume of the IP was completed to 500mL with additional lysis buffer and samples were rocked overnight on a wheel at 4°C. The day after, Dynabeads protein G (ThermoFisher Scientific) were washed 3 times in lysis buffer, resuspended 1:1 in lysis buffer and 50µL of beads were added to each Eppendorf tubes. Samples were rocked for 3 hours at 4°C to allow the coupling of the beads to the antibody. Afterwards, beads were collected and the supernatant was discarded. Beads were washed 4 times with washing buffer I (PBS supplemented with 1%NP-40, 0.5%NaDeoxycholate, 300mM NaCl, 1xRoche protease inhibitor and 25U/mL Superase-RNAse inhibitor). Beads were then resuspended in 100µL of RNAse free water and treated again with Turbo DNAseI for 30min at 37°C in a Thermomixer at 1100rpm. The input material was treated again with DNAseI. Then, beads were washed 4 times with washing buffer II (PBS supplemented with 1%NP-40, 0.5%NaDeoxycholate, 300mM NaCl, 10mM EDTA, 1xRoche protease inhibitor and 25U/mL Superase-RNAse inhibitor). Finally, RNA was eluted with elution buffer (100mM Tris-HCl pH 7.5, 50mM NaCl, 10mM EDTA, 100µg Proteinase K, 0.5% SDS for 1 hour at 55°C in a Thermomixer at 1100rpm, extracted using the RNA extraction kit (Ambion) and retro-transcribed into cDNA using the SuperScript III First-Strand Synthesis Super-Mix (Thermo Fisher Scientific). qPCRs were performed using SYBR GreenER qPCR SuperMix Universal (Invitrogen) and data expressed as relative enrichment to the respective input material.

The following primers were used for Real time analysis:

NC3 Fw 5’ GTAGGACTTGTGTGTCG 3’

NC3 Rv 5’ CTCACCGGTCGGCGATTC 3’

NC4 Fw 5’ GCAAGCAGAGAAGGCATAGCAG 3’

NC4 Rv 5’ ATTCTCCTTAGCTAGGAACCAGC 3’

hU1 Fw 5’ ATACTTACCTGGCAGGGGAG 3’

hU1 Rv 3’ CAGGGGGAAAGCGCGAACGCA 3’

A

B

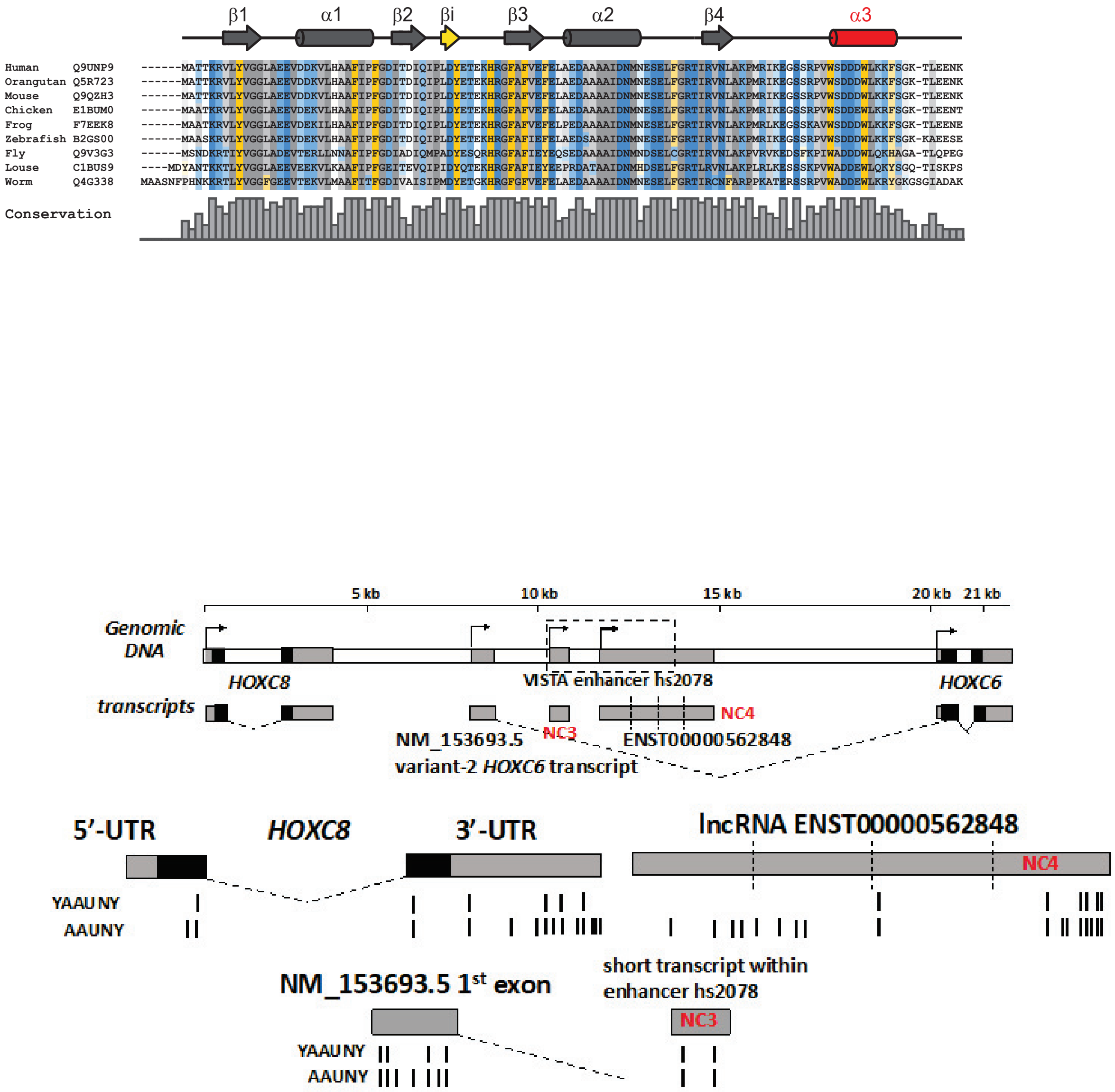

β1
α1
β2
βi
β3
α2
β4
α3
Human Q9UNP9
Orangutan Q5R723
Mouse Q9QZH3
Chicken E1BUM0
Frog F7EEK8
Zebrafish B2GS00
Fly Q9V3G3
Louse C1BUS9
Worm Q4G338
Conservation
5 kb
10 kb
15 kb
20 kb
21 kb
Genomic DNA
HOXC8
VISTA enhancer hs2078
HOXC6
transcripts
NC4
NC3
NM_153693.5
variant-2 HOXC6 transcript
ENST00000562848
5'-UTR
HOXC8
3'-UTR
lncRNA ENST00000562848
NC4
YAAUNY
AAUNY
NM_153693.5 1st exon
short transcript within enhancer hs2078
NC3
YAAUNY
AAUNY

C

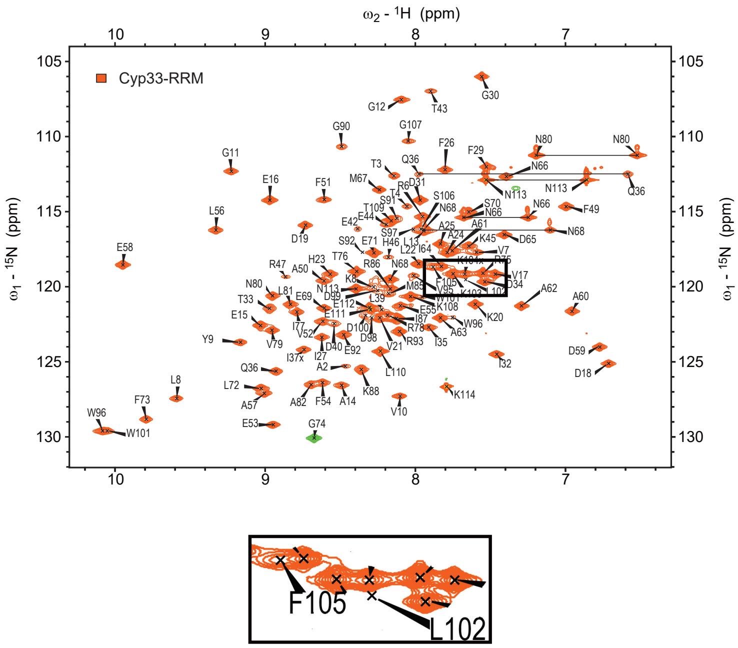

ω2 - 1H (ppm)
ω1 - 15N (ppm)
Cyp33-RRM
F105
L102

D

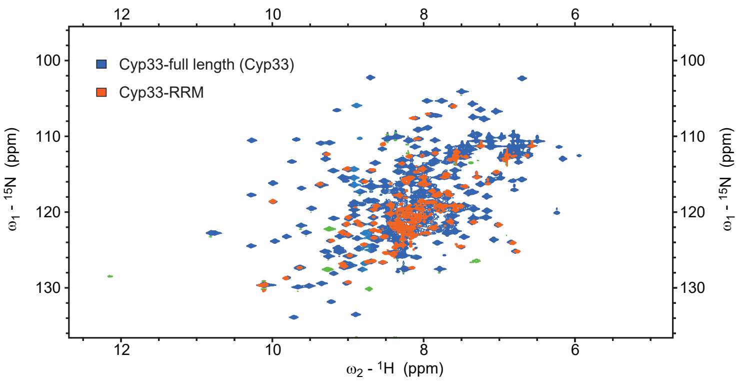

Cyp33-full length (Cyp33)
Cyp33-RRM
ω2 - 1H (ppm)
ω1 - 15N (ppm)

E

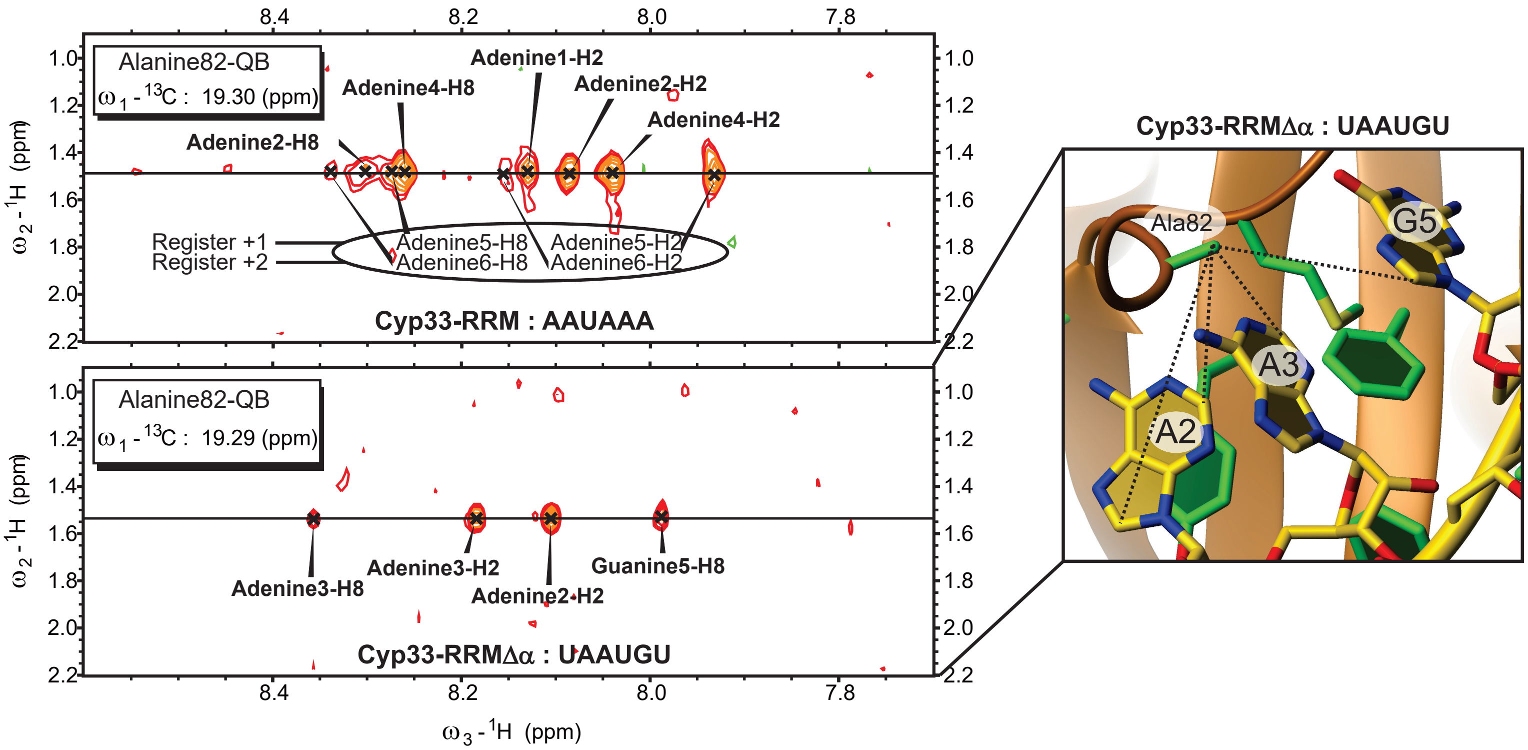

Alanine82-QB
ω1 - 13C : 19.30 (ppm)
Adenine4-H8
Adenine1-H2
Adenine2-H2
Adenine4-H2
Adenine2-H8
Register +1
Register +2
Adenine5-H8
Adenine6-H8
Adenine5-H2
Adenine6-H2
Cyp33-RRM : AAUAAA
Alanine82-QB
ω1 - 13C : 19.29 (ppm)
Adenine3-H8
Adenine3-H2
Adenine2-H2
Guanine5-H8
Cyp33-RRMΔα : UAAUGU
ω2 - 1H (ppm)
ω3 - 1H (ppm)
Cyp33-RRMΔα : UAAUGU
Ala82
G5
A3
A2

F

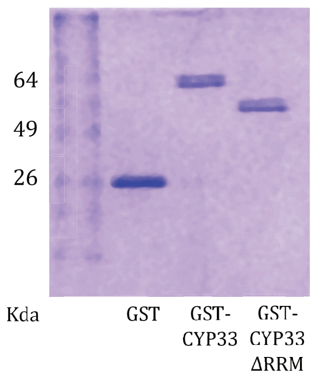

G

ACGUAUAAACGUUCUCCGCACGCAACUUAAUC
UCCCAAAUGCCCCAAUUCAUAUCCUCCGGC
AACUUAAUCCGAAUUAAGUCAAGAACGCGC
AAUCGCAAUUUUUAUAAACGCCCUUUUAUCC
ACAAUCAAUCCAAUCAUAACAUCCACGUUG
GAUUCACCUUCGGCGUAGUAAUCCGUUUCC
UAAUUGACUUUACUGCCUUGUAAUUUACAC
UUAAUCCUAACGGCCUUUUUGAAGACC
CGGGGUGGUAACUCAUUAAUCUCCUCGCCC
UUACAUCGCUUACUGUGUAAUCUAUGGCCC
ACCCAAUAUGCAACAUCGCAUCUCCGCACAGU
GGCUAAUGUCUCAUAGCGUGGUUGGGUCAC
AAACAACCUUGACCACAAUAUCGGGCGCUA
ACCUCUCACCCCAUCCUGUGAACAAUGUACUC
GUGCACAUGAAAACGAAUUCCUUAACCCAC
GUACAUCGCUUACUGUGGAAUCUAUGGCCG
UGAAUUGAAUACGGCCUCUUUCGACACGGA
AAAUUAUCUCAUUUAGUCGCGGAAUGUACC
GAUGAAAUGCCGGGCGAAGUCUUGUCUUGC
GCACAACCACGAUACAGAAUGUGUCCGUCC
ACUACAGCUCCUUCCCCAAUUACGGCGAUU
CAGUCCCUGACUAGAUGAUUAAUCGGGUCC
UGCCCAGAAAUAGUCGUCGCCAUAAUCGAC
UUCAUACCCCCAUUUUCGAAUAAUCACGCC
GUCAAACAAUCGAACGUGAUACAAGUGAUC
CAAUCAGAAAACGAACAACAGGCUCCUACG
AACGCUUCGUUUCUUUCCGCUGACAAUGAC
UCUCUCGUCAUCUCAUGCUAUAAUAAC
CAAGUAUGGGGAUUUCGAAUAAUGACGUC
CCCGCGCGAUCGAAUCGAUAUUAUCCGUCC
CUACGAAGAAUUGCUAUGUGCAUCUUGCUG
AGUACCCACACGAAAAAUUAACUUGCGUGC
CUGUGUGACCACUUAAAACGUUGCCAAAAC
AUACACACAUUACGCCAUUCGUGCUUAACA
CACCAGAGCACGUUACCGUAACCCGUAUCC
CAGUUUGGUUCGAUUAUAUUUUCUUCCUGC
UAAACCUAUCUGAUUAUGAACAUCUU
UUGAUCACUAGUAACCCUUCAGGCUACUCC
UUUAUGUUAUCCAACCAUAGAAAGCGGGGC
AGAAACAUACGAUUUGCGUCCGCAUCAG
UUGAUUUCCUACUGAGGUUGGCCCGCCUGA
CAAAACUACUAAGGAUAAAGCAUGAACCC
4UAUACAUUACGCAACAUUUCGCACUUCCAG
UUUCUCUAGAUAGGCACCACUUUCACUAAA
UUUGUAACAUCAACUUAUUUCCUCGAGUAC
UACGCUCAAAGUUGACUAUUGUUCCCUCCC

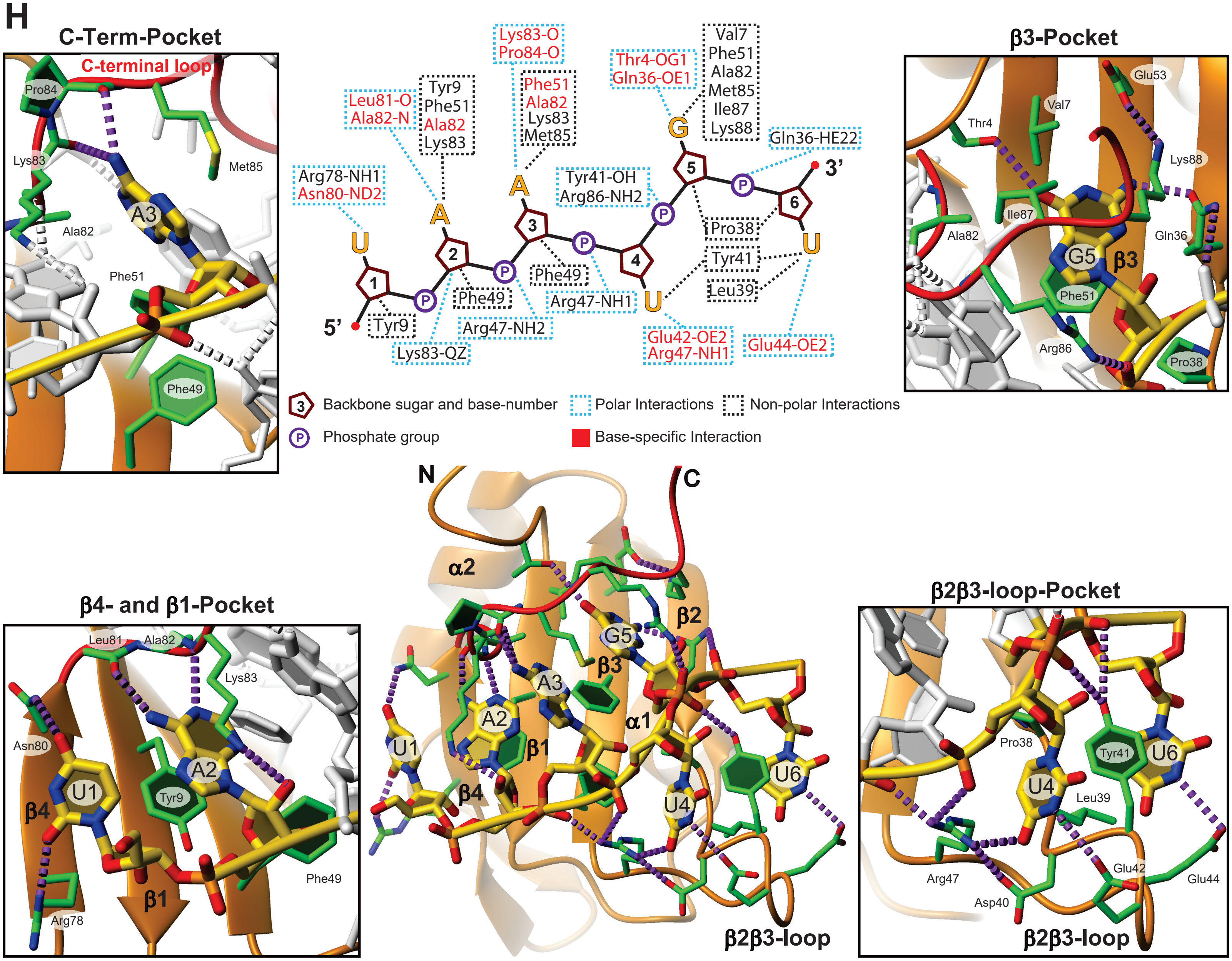

Figure S1: (A) Sequence alignment through Cyp33 orthologs. **(B)** Diagram of the genomic region between HOXC8 and HOXC6 showing several identified transcripts, and the location of the HOXC8 maintenance enhancer. The transcripts are shown as black or gray boxes for coding and non-coding sequences respectively; the ticks under the enlarged transcripts represent SELEX enriched motives. NC3 and NC4 are the sequences used in the UV-RIP experiment shown in Figure 2. **(C)** Assigned 2D [^{1}H,^{15}N] HSQC spectrum of free Cyp33-RRM. Close-up view showing the broadening of L102 and F105 cross-peaks. **(D)** Overlay of [^{1}H,^{15}N] HSQC spectra of Cyp33 full-length and RRM alone. The highly similar chemical shifts indicate independent tumbling of the cyclophilin domain and the RRM. **(E)** The multiple binding registers of Cyp33 RRM to the AAUAAA RNA. Representative strips of ω3-filtered, ω1-edited ^{13}C-resolved 3D NOESY spectra of samples containing either ^{13}C-Cyp33-RRM : ^{12}C-AAUAAA RNA (upper strip) or ^{13}C-Cyp33-RRMΔα : ^{12}C-UAAUGUCG RNA. The filtered nOe dimension ω3 shows cross-peaks between the protein proton of A82-QB to all nucleotides from three different registers (AAUAAA RNA) or one unique register (UAAUGUCG RNA). Contour levels are red and become orange for the most intense peaks. Close-up view of the structure shows the three bases involved in the nOes observed in the unique register of the Cyp33-RRMΔα : UAAUGUCG RNA complex. Spectra were visualized using the program SPARKY. **(F)** Electrophoretic separation of the GST tagged protein ligands used in the SELEX experiment. **(G)** 46 RNA sequences selected by the GST-CYP33 ligand after three rounds of selection. The AAU motif is the only triplet that was significantly enriched ($p < 0.01$). The YAAU and AAUY tetramers, the AAUNY and YAAUY pentamer and the YAAUYY and YAAUNY hexamer (in red box), all containing the AAU core sequence were also enriched ($p < 0.05$). These motifs were not enriched by binding to GST-CYP33ΔRRM used as control. **(H)** Structure of Cyp33 RRMΔα bound to the UAAUGUCG RNA. The protein secondary structure of the RRM is shown in orange, RNA and protein side-chains or backbone involved in RNA interactions are shown as sticks. Heavy atoms are shown in orange (P atoms), yellow (C atoms of RNA), green (C atoms of protein), red (O atoms) and blue (N atoms). H-bonds in magenta. A schematic representation of the protein-RNA interactions is shown.

A

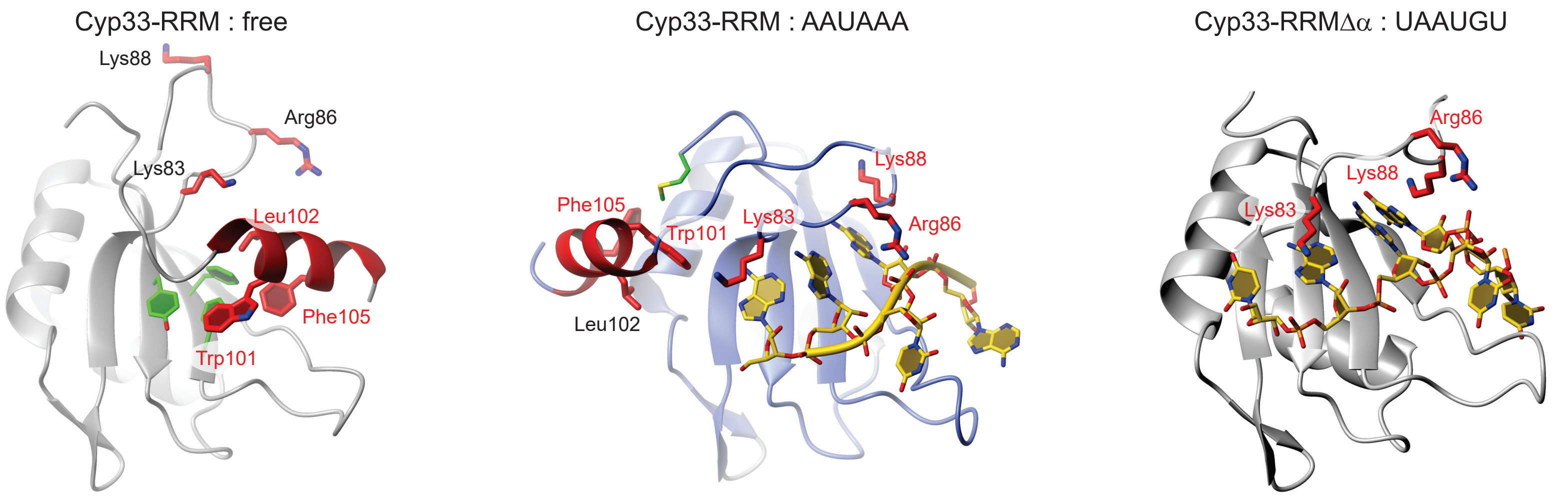

Mutant		***designed to:***
Cyp33-K83A	(Cyp33-K)	reduce RNA binding; reported ubiquitination site
Cyp33-R86A-K88A	(Cyp33-RK)	reduce RNA binding
Cyp33-K83A-R86A-K88A	(Cyp33-KRK)	reduce RNA binding
Cyp33-W101-L102-F105A	(Cyp33-WLF)	enhance RNA binding

B

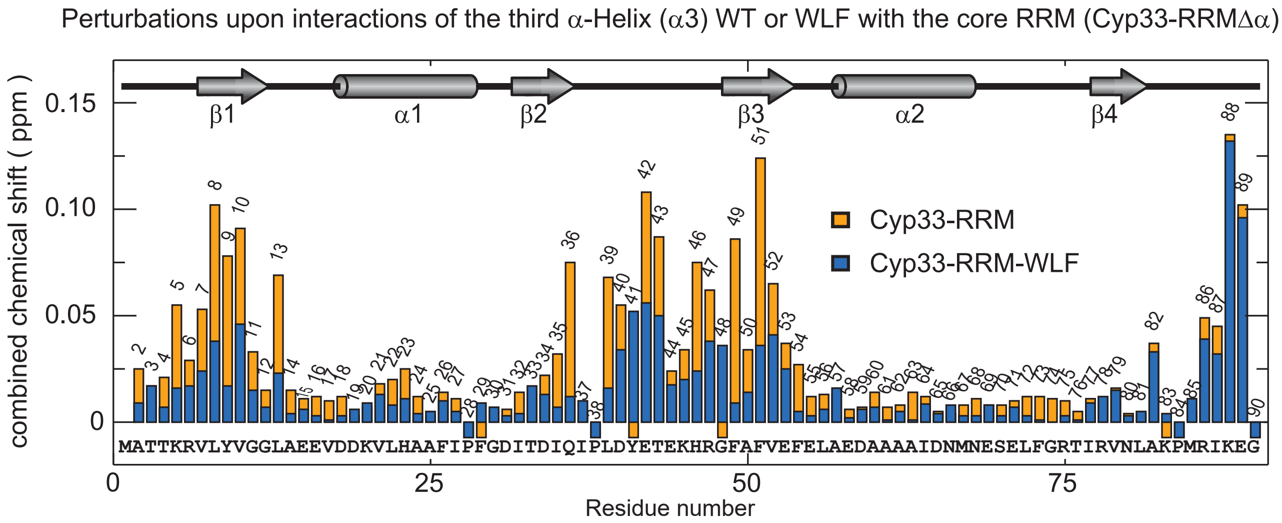

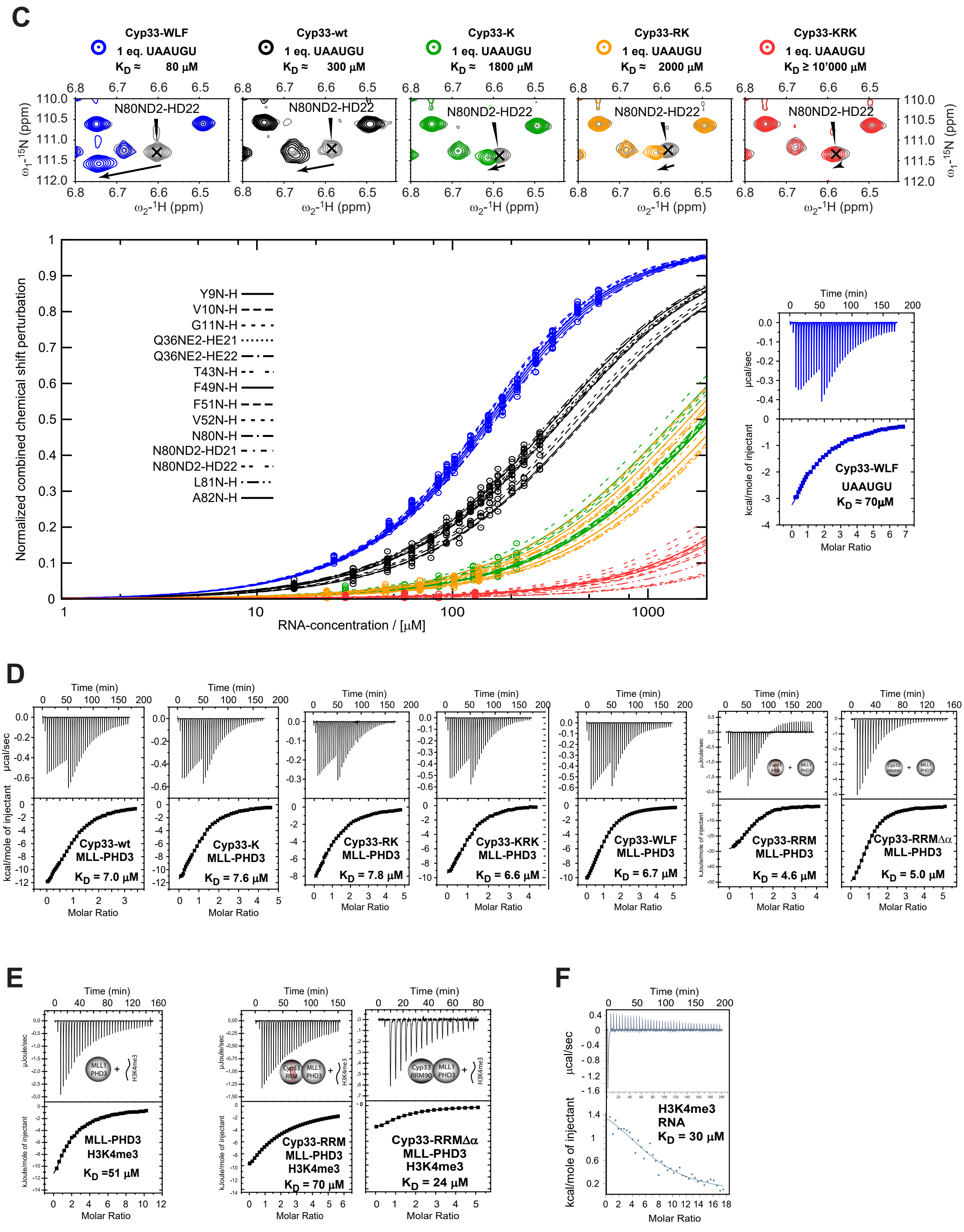

C
Cyp33-WLF
1 eq. UAAUGU
K_D ≈ 80 μM
Cyp33-wt
1 eq. UAAUGU
K_D ≈ 300 μM
Cyp33-K
1 eq. UAAUGU
K_D ≈ 1800 μM
Cyp33-RK
1 eq. UAAUGU
K_D ≈ 2000 μM
Cyp33-KRK
1 eq. UAAUGU
K_D ≥ 10'000 μM
N80ND2-HD22
ω1-15N (ppm)
ω2-1H (ppm)
Normalized combined chemical shift perturbation
RNA-concentration / [μM]
Y9N-H
V10N-H
G11N-H
Q36NE2-HE21
Q36NE2-HE22
T43N-H
F49N-H
F51N-H
V52N-H
N80N-H
N80ND2-HD21
N80ND2-HD22
L81N-H
A82N-H
Time (min)
μcal/sec
kcal/mole of injectant
Molar Ratio
Cyp33-WLF
UAAUGU
K_D ≈ 70μM
D
Cyp33-wt
MLL-PHD3
K_D = 7.0 μM
Cyp33-K
MLL-PHD3
K_D = 7.6 μM
Cyp33-RK
MLL-PHD3
K_D = 7.8 μM
Cyp33-KRK
MLL-PHD3
K_D = 6.6 μM
Cyp33-WLF
MLL-PHD3
K_D = 6.7 μM
Cyp33-RRM
MLL-PHD3
K_D = 4.6 μM
Cyp33-RRMΔα
MLL-PHD3
K_D = 5.0 μM
E
MLL-PHD3
H3K4me3
K_D =51 μM
Cyp33-RRM
MLL-PHD3
H3K4me3
K_D = 70 μM
Cyp33-RRMΔα
MLL-PHD3
H3K4me3
K_D = 24 μM
kJoule/mole of injectant
μJoule/sec
F
H3K4me3
RNA
K_D = 30 μM

G

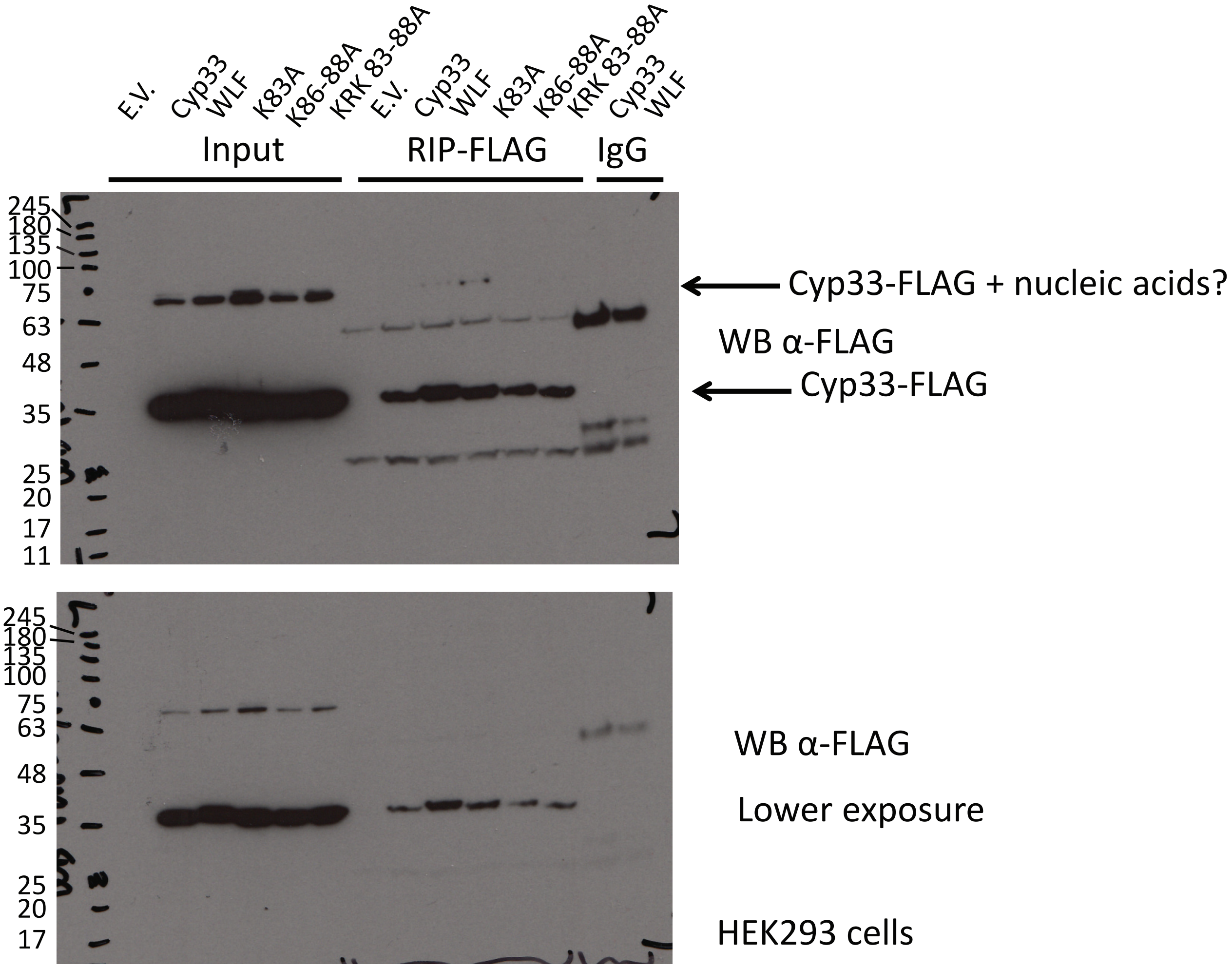
E.V.
Cyp33 WLF
K83A
K86-88A
KRK 83-88A
E.V.
Cyp33 WLF
K83A
K86-88A
KRK 83-88A
Cyp33 WLF
Input
RIP-FLAG
IgG
245
180
135
100
75
63
48
35
25
20
17
11
Cyp33-FLAG + nucleic acids?
WB α-FLAG
Cyp33-FLAG
245
180
135
100
75
63
48
35
25
20
17
WB α-FLAG
Lower exposure
HEK293 cells

H

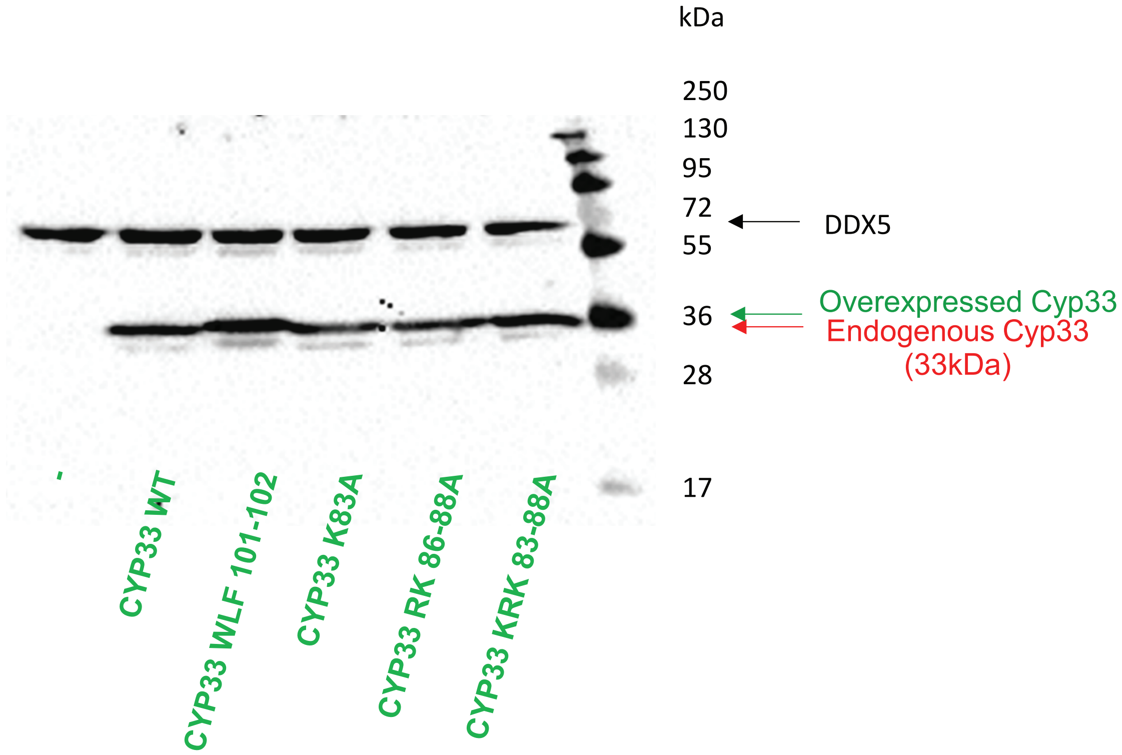
kDa
250
130
95
72
55
36
28
17
DDX5
Overexpressed Cyp33
Endogenous Cyp33
(33kDa)
-
CYP33 WT
CYP33 WLF 101-102
CYP33 K83A
CYP33 RK 86-88A
CYP33 KRK 83-88A

I

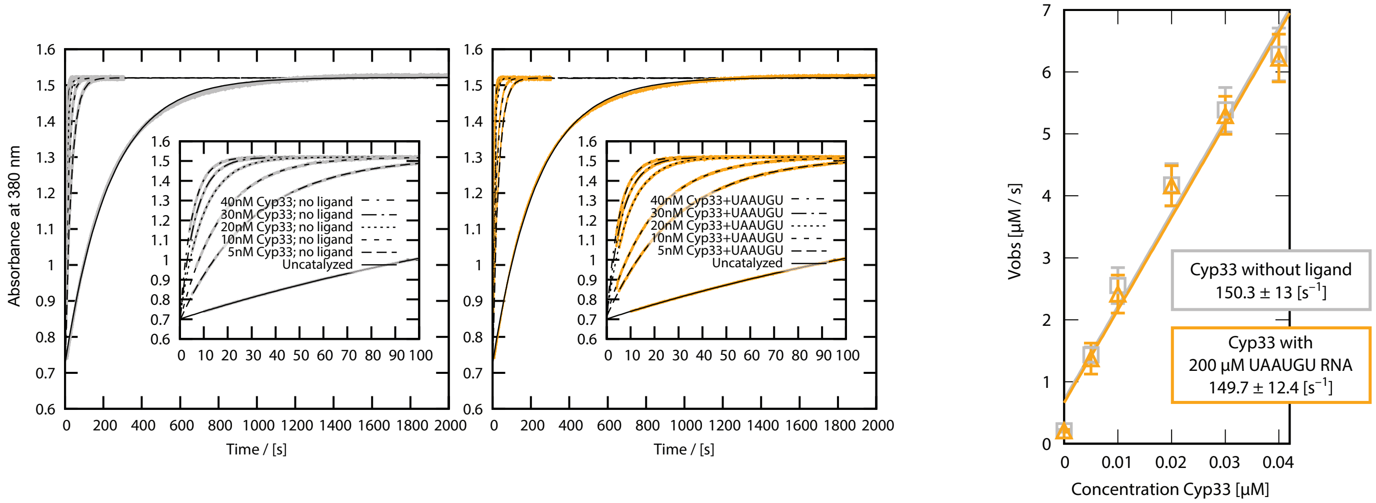

Figure S2: Design of the Cyp33 RNA binding mutants. (A) Residues that are mutated in this study are shown on the structure of Cyp33 RRM free and bound to RNA. **(B)** Chemical shift differences observed between Cyp33 RRMΔα and either Cyp33 RRM WT (in orange) or WLF (in blue). **Affinity measurements. (C)** Kd values obtained by NMR for WT and mutated versions of Cyp33 RRM with the UAAUGU RNA. **(D)** Kd values obtained by ITC for WT and mutated versions of Cyp33 RRM with the MLL-PHD3 protein. **(E)** Kd values obtained by ITC for MLL-PHD3 and the H3K4me3 peptide in the absence or in the presence of Cyp33 RRM or Cyp33 RRMΔα. **(F)** Kd value obtained by ITC for the H3K4me3 peptide in the presence of the 4 UAAUGU repeats RNA. **Western blots of WT and mutated Cyp33 proteins after transfection. (G)** Western blot of WT and mutated Cyp33 proteins after 24h of expression in HEK293 cells and before UV-RIP experiments. **(H)** Western blot of WT and mutated Cyp33 proteins after 48h of expression in HEK293T cells and before the RT-qPCR analysis. The nuclear DDX5 protein was used as a control. **(I)** Representative kinetic traces and PPIase turnover numbers of Cyp33 in free state (gray) and bound to UAAUGU RNA (orange, 200mM RNA). Error bars were obtained from five (Cyp33 free) and three (Cyp33 RNA bound) independent measurements.

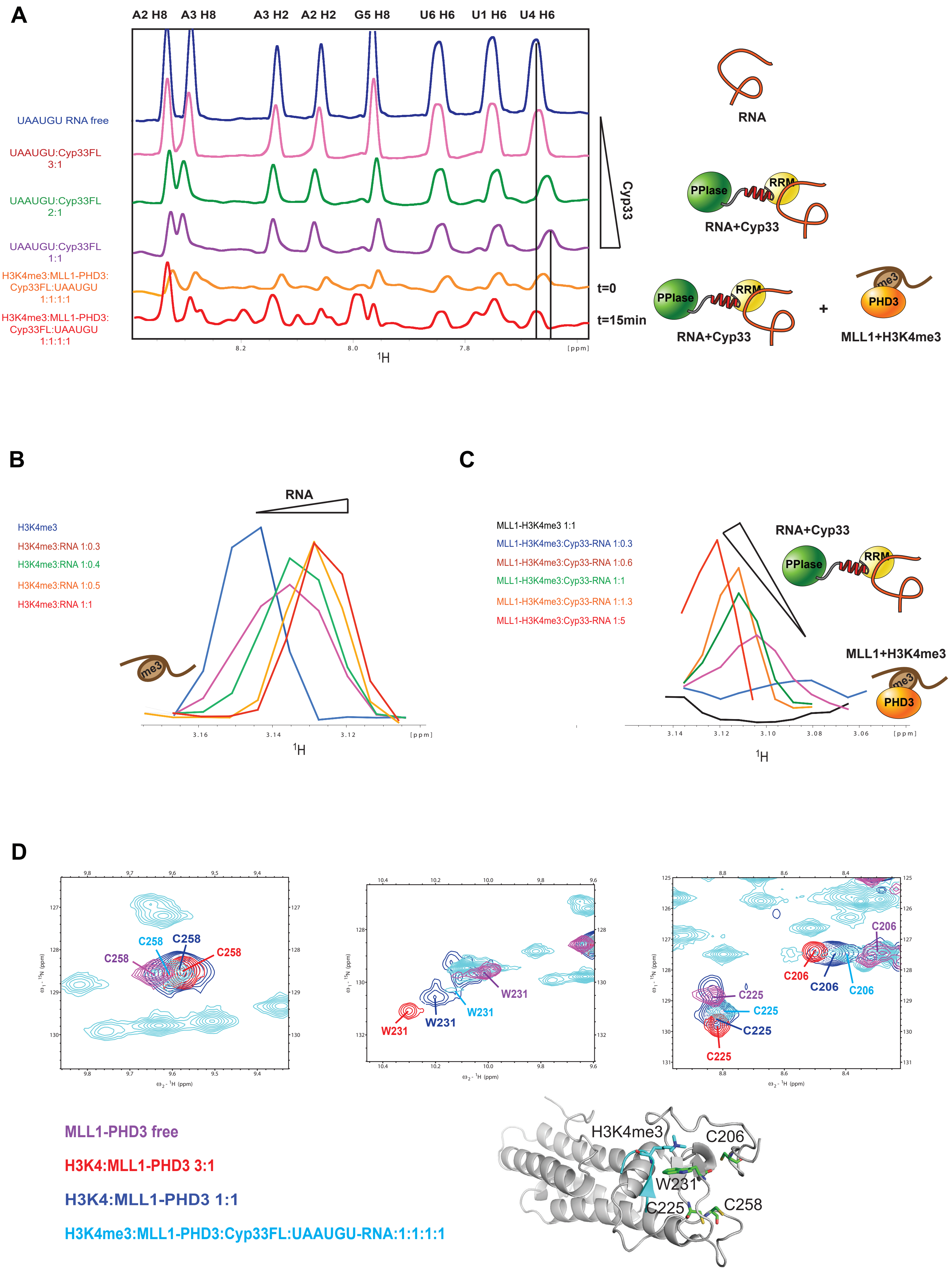
A
A2 H8
A3 H8
A3 H2
A2 H2
G5 H8
U6 H6
U1 H6
U4 H6
UAAUGU RNA free
UAAUGU:Cyp33FL 3:1
UAAUGU:Cyp33FL 2:1
UAAUGU:Cyp33FL 1:1
H3K4me3:MLL1-PHD3: Cyp33FL:UAAUGU 1:1:1:1
H3K4me3:MLL1-PHD3: Cyp33FL:UAAUGU 1:1:1:1
Cyp33
t=0
t=15min
8.2
8.0
7.8
[ppm]
1H
RNA
PPlase
RRM
RNA+Cyp33
me3
PHD3
MLL1+H3K4me3
B
RNA
H3K4me3
H3K4me3:RNA 1:0.3
H3K4me3:RNA 1:0.4
H3K4me3:RNA 1:0.5
H3K4me3:RNA 1:1
3.16
3.14
3.12
[ppm]
1H
C
MLL1-H3K4me3 1:1
MLL1-H3K4me3:Cyp33-RNA 1:0.3
MLL1-H3K4me3:Cyp33-RNA 1:0.6
MLL1-H3K4me3:Cyp33-RNA 1:1
MLL1-H3K4me3:Cyp33-RNA 1:1.3
MLL1-H3K4me3:Cyp33-RNA 1:5
RNA+Cyp33
MLL1+H3K4me3
3.14
3.12
3.10
3.08
3.06
[ppm]
1H
D
C258
W231
C206
C225
MLL1-PHD3 free
H3K4:MLL1-PHD3 3:1
H3K4:MLL1-PHD3 1:1
H3K4me3:MLL1-PHD3:Cyp33FL:UAAUGU-RNA:1:1:1:1
H3K4me3
C206
W231
C225
C258

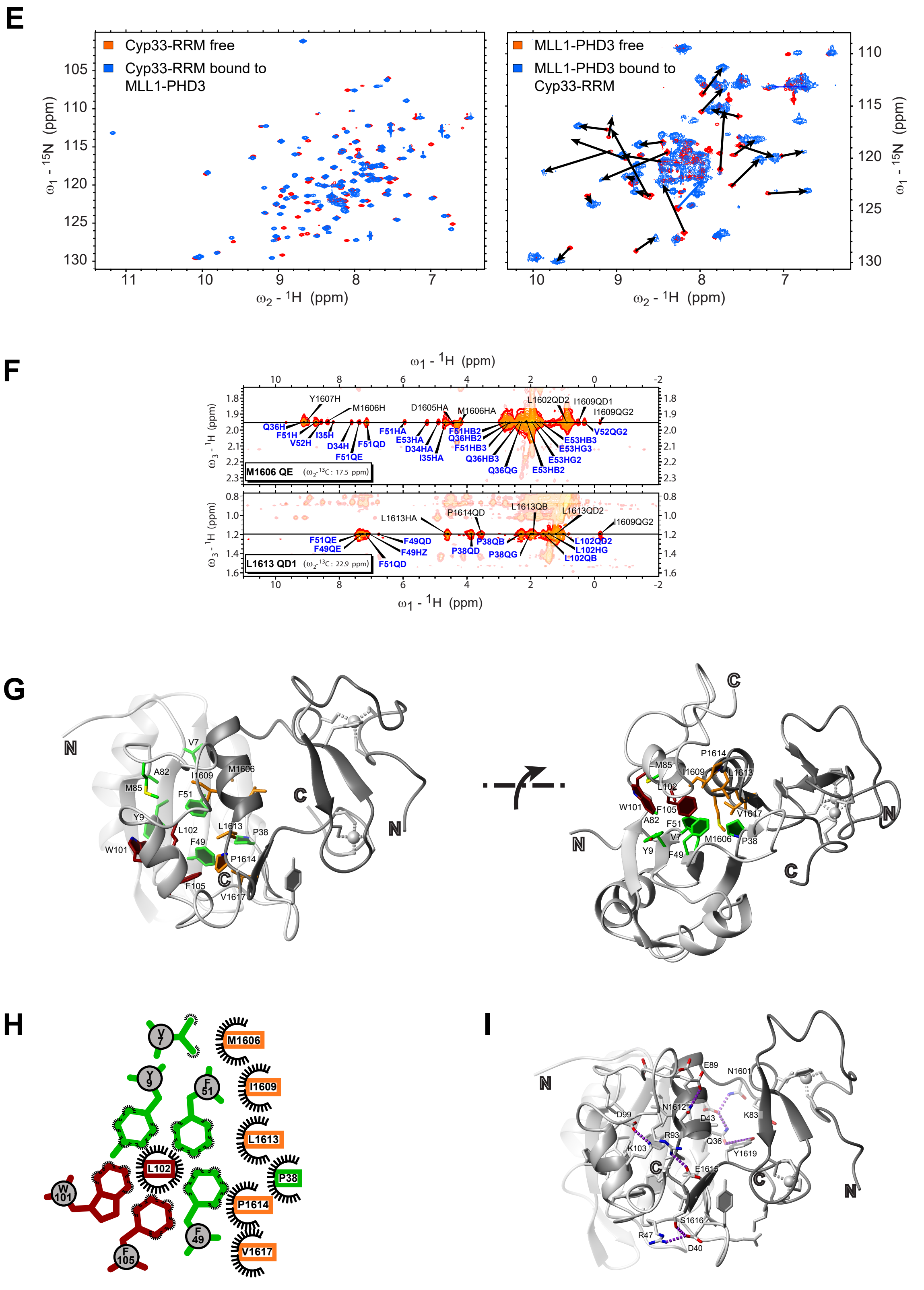
E
Cyp33-RRM free
Cyp33-RRM bound to MLL1-PHD3
MLL1-PHD3 free
MLL1-PHD3 bound to Cyp33-RRM
ω1 - 15N (ppm)
ω2 - 1H (ppm)
F
ω1 - 1H (ppm)
ω3 - 1H (ppm)
M1606 QE (ω2-13C: 17.5 ppm)
L1613 QD1 (ω2-13C: 22.9 ppm)
Y1607H
M1606H
D1605HA
M1606HA
L1602QD2
I1609QD1
I1609QG2
Q36H
F51H
V52H
I35H
D34H
F51QD
F51QE
F51HA
E53HA
D34HA
I35HA
F51HB2
Q36HB2
F51HB3
Q36HB3
Q36QG
E53HB3
E53HG3
E53HG2
E53HB2
V52QG2
L1613HA
P1614QD
L1613QB
L1613QD2
I1609QG2
F51QE
F49QE
F51QD
F49QD
F49HZ
P38QD
P38QB
P38QG
L102QD2
L102HG
L102QB
G
N
C
V7
A82
M85
I1609
M1606
F51
Y9
L102
L1613
P38
F49
W101
P1614
F105
V1617
M85
P1614
I1609
L1613
L102
W101
F105
A82
F51
V7
V1617
M1606
P38
Y9
F49
H
V7
M1606
Y9
F51
I1609
L1613
W101
L102
P38
P1614
F105
F49
V1617
I
E89
N1601
N1612
D99
D43
K83
R93
Q36
K103
Y1619
E1615
S1616
R47
D40

Fig. S3: Interaction of Cyp33 full length (FL) bound to RNA with MLL1 PHD3 leads to the release of the H3K4me3 peptide.
(A) Changes in the chemical shift of RNA protons upon addition of increasing concentrations of Cyp33FL and then of the MLL1 PHD3/H3K4me3 complex to the Cyp33FL: UAAUGU complex at t=0 (yellow) and t=15min (orange). **(B)** Changes in the chemical shift of H3K4me3 upon addition of increasing concentrations of RNA. **(C)** Changes in the chemical shift of H3K4me3 upon addition of increasing concentrations of Cyp33-RNA 1:1 complex to MLL1-H3K4me3 1:1 complex. **(D)** Overlay of 1H-15N HSQC spectra recorded with MLL1 PHD3 in the free form (magenta), in the presence of increasing concentrations of H3K4me3 and finally after addition of the Cyp33FL:RNA complex (cyan). Crystal structure of MLL1 PHD3-BRD complexed with the H3(1-9)K4me3 peptide in cyan (Wang et al., 2010, reference 13). **(E)** Overlay of [^{1}H,^{15}N] HSQC spectra of Cyp33-RRM and MLL1-PHD3 in their free state and complexed. **(F)** Two representative strips of a ^{13}C-resolved 3D NOESY spectrum of a sample containing ^{13}C-Cyp33-RRM : ^{13}C-MLL-PHD3 for which the nOe dimension ω1 shows multiple intra- (black) and inter-molecular (blue) cross-peaks. Spectra were visualized using the program SPARKY. **(G)** Most representative conformer of Cyp33-RRM (gray) : MLL-PHD3 (black) complex in ribbon representation. Sidechains forming the massive hydrophobic core at the molecular interface are shown in green (Cyp33-RRM-core domain), red (Cyp33-RRM-alpha3) and orange (MLL-PHD3). **(H)** Schematic interaction network of hydrophobic intermolecular core using the same color code as in panel C. **(I)** ribbon representation as in panel C. Polar sidechains shielding the hydrophobic intermolecular core from the solvent are displayed with colored nitrogen atoms (blue), oxygen atoms (red) and potential salt-bridges or hydrogen bonds are shown with dashed purple lines.

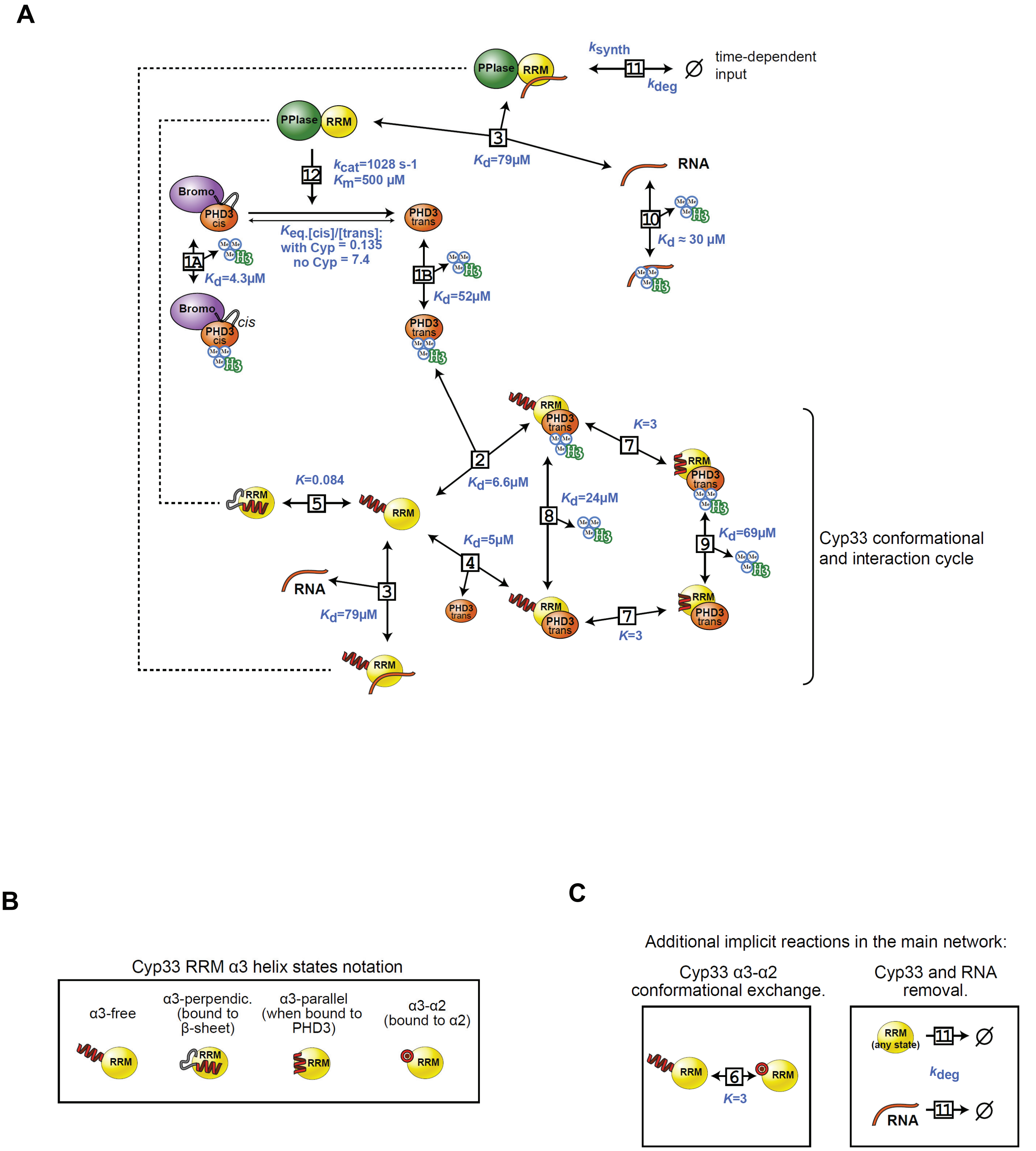
A
PPIase
RRM
ksynth
11
kdeg
time-dependent input
3
Kd=79μM
RNA
12
kcat=1028 s-1
Km=500 μM
Bromo
PHD3 cis
PHD3 trans
Keq.[cis]/[trans]:
with Cyp = 0.135
no Cyp = 7.4
1A
Kd=4.3μM
1B
Kd=52μM
10
Kd ≈ 30 μM
cis
H3
Me
K=3
7
2
Kd=6.6μM
K=0.084
5
8
Kd=24μM
9
Kd=69μM
4
Kd=5μM
Cyp33 conformational and interaction cycle
B
Cyp33 RRM α3 helix states notation
α3-free
α3-perpendic. (bound to β-sheet)
α3-parallel (when bound to PHD3)
α3-α2 (bound to α2)
C
Additional implicit reactions in the main network:
Cyp33 α3-α2 conformational exchange.
6
K=3
Cyp33 and RNA removal.
(any state)
kdeg

D

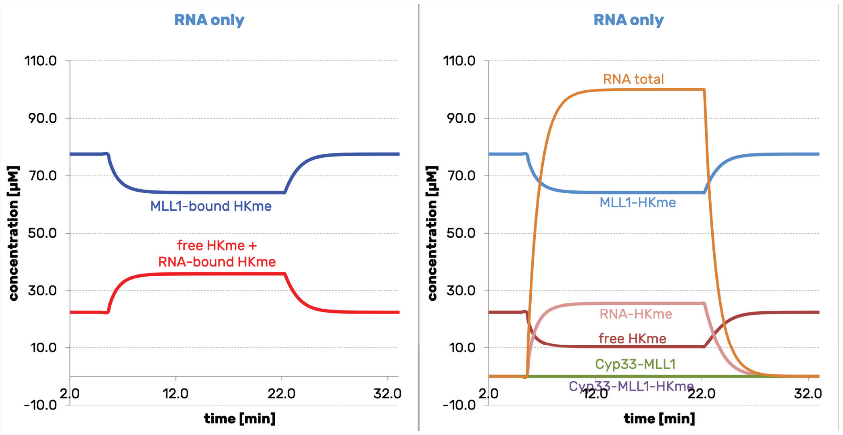

E

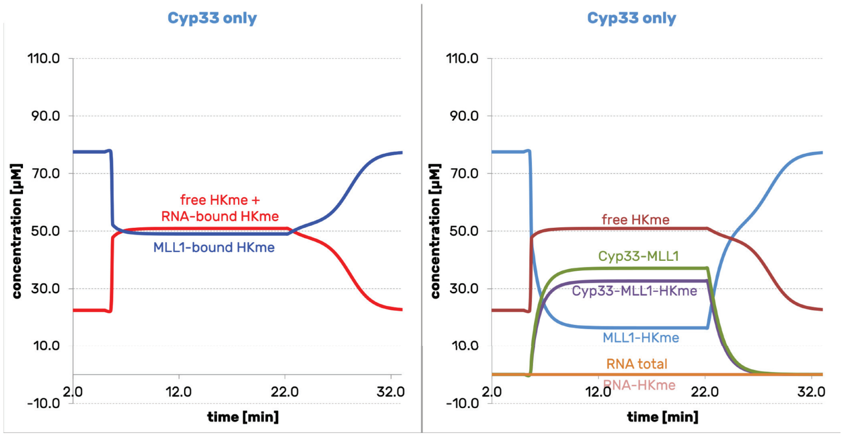

F

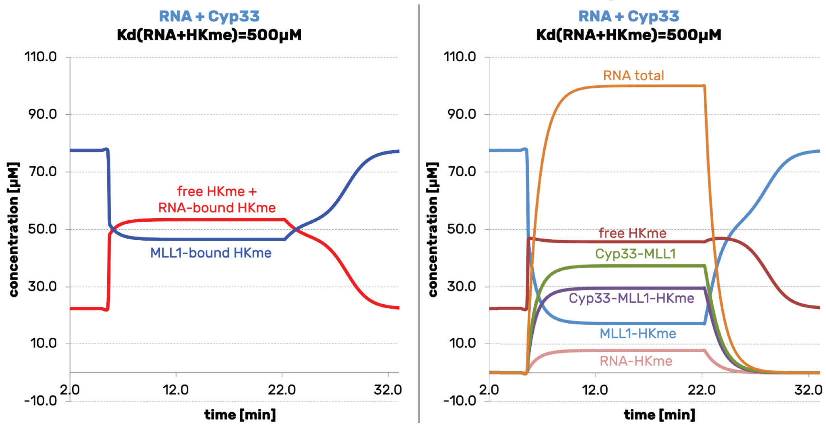

Figure S4. Schema of reactions in the reconstructed ODE network and additional ODE network simulations.
(A) Reactions are shown as solid arrows with specific reaction IDs. Key constants for each reaction indicated in blue text. For unimolecular reactions the equilibrium constants K larger than 1 indicate the balance in favour of reaction products (the right side of the arrow). Details on each reaction and constant are in Supplementary Table S2. Molecular species colours correspond to those in Figure 6. Dashed lines indicate equivalent species between remote parts of the network. Specifically, all Cyp33-RRM species in the bottom part of the network implicitly include full Cyp33 protein with the PPIase domain, although this domain is explicitly shown only in the two top reactions in the network. Bromo domain of MLL1 is explicitly shown only in context of the PHD3-cis-proline species, and is assumed to be implicitly present in all PHD3-trans-proline species. **(B)** Graphical notation for the four conformational states of the Cyp33-RRM α3 helix used in the main scheme. **(C)** Additional reactions which are included in the ODE model, but are omitted in the main model scheme for simplicity. Simulations done under the same conditions as Figure 6A, with the following differences: **(D)** Effect of RNA alone – Cyp33 was not recruited during RNA transcription; **(E)** Effect of Cyp33 alone – RNA was not transcribed; **(F)** Both RNA and Cyp33 were generated, but the RNA-H3K4me KD was set to 500 µM (17-fold weaker affinity than the experimentally determined 30 µM) – to illustrate robustness of the system behavior.Simulations show equilibrium populations of different species in the target system, as simulated by the developed ODE model encompassing all underlying reactions. At t=0 min, the initial system is equilibrated with only MLL1 and H3K4me at 100 µM each. At t=5.6 min RNA transcription is triggered, simultaneously recruiting Cyp33 to the system – with each species reaching 100 µM concentration at its maximum – thus giving equimolar concentrations for all 4 components of the system (MLL1, H3K4me, RNA, Cyp33). Simulations in (D) and (E) show cases when only RNA and only Cyp33 were dynamically added to the system. At t=22 min, RNA transcription is turned off, allowing gradual removal of RNA and Cyp33 from the system. Left panels show the balance between the “active” (blue trace) and “repressive” (red trace) states of H3K4me. The “active” state combines the binary MLL1-H3K4me and tertiary Cyp33-MLL1-H3K4me complexes. The “repressive” state combines the free H3K4me and RNA-bound H3K4me. Right panels show the corresponding time-resolved dynamics for each of the above species separately.

Supplementary Table 1. Summary table of affinities, catalytic PPIase efficiency of Cyp33 and the structural conformations of the third helix in the RRM of Cyp33 (α_3).

Receptor	Ligand	KD / [µM][a]	Kcat / $\left[\frac{1}{s}\right]$	Orientation $a3$ [b]
Cyp33	No Ligand	NA	150.3 ±13.0	crosswise
Cyp33-WLF101-105A	UAAUGU	79±7 [d], 71 [d]	NM	free
Cyp33	UAAUGU	319±54 [c]	149.7 ±12.4	exposed
Cyp33-K83A	UAAUGU	1800±300 [c]	NM	crosswise
Cyp33-RK86-88A	UAAUGU	2060±450 [c]	NM	crosswise
Cyp33-KRK83-88A	UAAUGU	≥ 10'000 [c]	NM	crosswise
Cyp33	MLL1-PHD3	7.0 [d]	NM	parallel
Cyp33-K83A	MLL1-PHD3	7.6 [d]	NM	parallel
Cyp33-RK86-88A	MLL1-PHD3	7.8 [d]	NM	parallel
Cyp33-KRK83-88A	MLL1-PHD3	6.6 [d]	NM	parallel
Cyp33-WLF101-105A	MLL1-PHD3	6.7 [d]	NM	free
Cyp33-RRM	MLL1-PHD3	4.6 [d]	NA	parallel
Cyp33-RRMΔα	MLL1-PHD3	5.0 [d]	NA	deleted
RNA $(UAAUGU)_4$	H3K4me3	30.0 [d]	NA	NA
MLL1-PHD3	H3K4me3	51.6 [d]	NA	NA
Cyp33-RRM : MLL1-PHD3	H3K4me3	69.0 [d]	NA	parallel
Cyp33-RRMΔα : MLL1-PHD3	H3K4me3	23.2 [d]	NA	deleted

a Dissociation Constant (K_D)
b Position of the third α-helix in the RRM of Cyp33 relative to the β-sheet ($\alpha3$)
c Affinity measured by NMR
d Affinity measured by ITC
NA Not applicable
NM Not measured

Supplementary Table S2. Parameter values and species concentrations used in the simulations.

- The network simulation conclusions in the paper refer only to equilibrium states, defined by thermodynamic constants. Kinetic constants are estimated primarily for ODE model definition purposes and, within the simulation timescales used in the paper, do not affect the network equilibrium states.
    - Unless indicated otherwise the bimolecular kinetic k_{on} constants are assumed to be 10^2 slower than the absolute diffusion limit – to approach the unbiased diffusion-limited k_{on} (*70*). The diffusion limits are estimated based on spatial dimensions of corresponding molecules.
- "Experimental" – refers to measurements performed in this study. K_D constants measured by ITC or NMR at 25ºC (298.15K).
- Main simulations presented in the paper were done at equimolar 100 μM of MLL-PHD3, H3K4me (present from the start of simulation) and 100 μM RNA and Cyp33 being first dynamically recruited and then removed during simulation.

#	Reaction	Parameter values	Comments
1A	PHD3 (**cis-proline** Bromo) binding to H3K4me	K_d = 4.3 μM<br>Wang et al. 2010 (*13*)<br>k_{on} = 8.1*10^7 M^{-1} s^{-1}<br>k_{off} = K_d * k_{on}	
1B	PHD3 (**trans-proline** Bromo) binding to H3K4me	K_d = 52 μM<br>(experimental)<br><br>k_{on} = 8.1*10^7 M^{-1} s^{-1}<br>k_{off} = K_d * k_{on}	
2	Cyp33 binding to [PHD3-H3K4me] complex	K_d = 6.6 μM<br>(from thermodynamic cycle – see right)<br><br>k_{on} = 7.5*10^7 M^{-1} s^{-1}<br>k_{off} = K_d * k_{on}	- Cyp33 can be in "α3-free" and "α3-α2" states (affinity is the same for both).<br>- K_d = 6.6 μM is derived via free energies (ΔG) in the 4-reaction thermodynamic cycle, where ΔG=–RT*ln(K_d) values for 3 reactions (Cyp33+PHD3, PHD3+H3K4me and [Cyp33-PHD3]+H3K4me binding) are known:<br>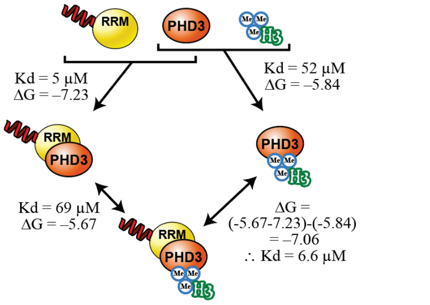
3	RNA binding to Cyp33	K_d = 79 μM<br>(experimental)<br><br>k_{on} = 7.6*10^7 M^{-1} s^{-1}<br>k_{off} = K_d * k_{on}	- Reaction occurs only if beta-sheet is free (not obstructed by α3).<br>- Affinity to RNA is the same when Cyp33 is in "α3-free" and "α3-α2" states (i.e. in both states a3-helix is away from beta-sheet).<br>- K_d = 79 μM is taken based on the Cyp33WLF mutant (assuming this is close to the "true" K_d of beta-sheet-RNA binding).<br>- Cyp33 and RNA longest dimensions (for diffusion-limited k_{on} calc) are taken as: 44 Å and 32.6 Å (based on Cyp33RRM90-UAAUGUCG PDB structure)

4	Free PHD3 binding to free Cyp33	K_d = 5 μM (experimental)<br><br>$k_{on} = 7.6*10^7\ M^{-1}\ s^{-1}$<br>$k_{off} = K_d * k_{on}$	- Cyp33 can be in "α3-free" and "α3-α2" states (affinity is the same for both).
5	α3 transition from **free** state to "**α3-perpendicular**" (bound to beta-sheet) state	$k_{on} = 1.3*10^7\ s^{-1}$<br>$k_{off} = 1.1*10^6\ s^{-1}$<br><br>$K_{eq}$ = 0.084	- k_{on} estimated from "3D-diffusion-to-capture" mechanism (the time it takes a protein to find its target by simple 3D diffusion) (*71*).<br>$\tau_{3D} = V / (D * a)$. $k_{on} = 1/\tau_{3D}$<br>V, D, a – search volume, diffusion coefficient, linear size of the target. Since α3-helix (26 Å-long) is tethered with one end, it is assumed that the search occurs in a volume of radius defined by helix length ($V=4/3 * \pi * (26\ Å)^3$. The linear size of the target surface is set equal to helix length (a = 26 Å). Diffusion coefficient is assumed to be same as for helix free in solution ($1.9E\text{-}06\ cm^2\ s^{-1}$). The fact that helix is attached to the protein with one end is neglected. Two main consequences of this, to a rough approximation, should balance one another: helix should move (diffuse) slower than free in solution, and at the same time it should find the target faster, since the search space is reduced.<br><br>- k_{off} is estimated based as follows:<br>(1) the equilibrium fraction of α3-perp (bound to beta-sheet) state should explain the Cyp33-RNA K_d enhancement 319 > 79 μM upon helix "removal" in WLF mutant (i.e. 75% of Cyp33 should be in α3-perp state at equilibrium)<br>(2) k_{on} rates for α3-perp and α3-α2 formation should be same (the diffusion search occurs in same volume)<br>(3) k_{off} rate for α3-perp state is 4 times slower than k_{off} of α3-α2 state (beta-sheet-bound state is expected to be more stable) – as judged based on relative interaction interfaces between the molecules.
6	α3 transition from **α3-free** state to "**α3-α2**" (bound to **α2**) helix	$k_{on} = 1.3*10^7\ s^{-1}$<br>$k_{off} = 4*(1.1*10^6)\ s^{-1}$<br>$k_{on,\ \alpha3\text{-}\alpha2} = k_{on,\ \alpha3\text{-perp}}$<br>$k_{off,\ \alpha3\text{-}\alpha2} = 4*k_{off,\ \alpha3\text{-perp}}$<br>$K_{eq} \approx 3$	- k_{on} – same as for reaction #5<br>- k_{off} – 4 times slower than for reaction #5 (α3-perp)
7	transition **α3-free** to "**α3-parallel**"	$k_{on} = 1.3*10^7\ s^{-1}$<br>$k_{off} = 4*(1.1*10^6)\ s^{-1}$<br>$k_{on,\ \alpha3\text{-par}} = k_{on,\ \alpha3\text{-perp}}$<br>$k_{off,\ \alpha3\text{-par}} = 4*k_{off,\ \alpha3\text{-perp}}$<br>$K_{eq} \approx 3$	- Requires that PHD33 domain is bound to beta-sheet of Cyp33<br>- k_{on} and k_{off} are estimated in same way as for reaction #6 (transition from α3-free to α3-α2 state).<br>- k_{on} and k_{off} rates for this α3 transition are set the same in binary (Cyp33-PHD3) and tertiary (Cyp33-PHD3-H3K4me) complexes.
8	H3K4me binding to [Cyp33-PHD3]	K_d = 24 μM (experimental)	Reaction occurs only if Cyp33-α3 is NOT in parallel orientation

	complex (with **α3-free** or **α3-α2**)	k_{on} = 9.8*10^7 M^{-1} s^{-1}<br>k_{off} = K_d * k_{on}	
9	H3K4me binding to [Cyp33-PHD3] complex (with **α3-parallel**)	K_d = 69 μM (experimental)<br><br>k_{on} = 9.8*10^7 M^{-1} s^{-1}<br>k_{off} = K_d * k_{on}	Reaction occurs only if Cyp33-**α3** IS in parallel orientation
10	RNA binding to H3K4me	K_d = 30 μM (experimental)<br>k_{on} = as in reaction #3<br>k_{off} = K_d * k_{on}	
11	RNA transcription, Cyp33 recruitment, RNA and Cyp33 removal.	k_{synth} = 1.5 s^{-1}<br>k_{deg} = 0.015 s^{-1}	k_{synth} – approximate RNAP II transcription speed of 10-20 nt/s (*72*), taking into account the slow transcription initiation phase.<br>k_{deg} removal rate is set 100-fold lower as k_{synth}, to reach 100 μM total RNA at equilibrium – thereby approaching equimolar concentrations of all main components of the system (RNA, Cyp33, H3K4me, PHD3), as in NMR experiments.
12	Cis-trans proline isomerization	In presence of Cyp33<br>*K*eq = [cis]/[trans] = 0.135<br><br>In absence of Cyp33, *K*eq = [cis]/[trans] = 1/0.135 = 7.4<br><br>PPIase catalyzed:<br>k_{cat} ≥ 1027.5 s^{-1} (experimental)<br><br>K_m ≈ 500 μM (mean value from BRENDA database for the enzyme EC 5.2.1.8)	In presence of Cyp33, *K*eq = [cis]/[trans] = 0.135 is assumed – based on the reported data for His-Pro bonds (*73*).<br><br>In absence of Cyp33 – based on the structural data (*13*) the cis isomer is predicted to be preferred. Here this is modelled as a reverse equilibrium of cis/trans isomer ratio from 0.135 to 1/0.135.<br><br>k_{cat} turnover number measured here (150 s^{-1} at 4ºC) was scaled to 25ºC to match the temperature of all other constants using the Q10 temperature coefficient of 2.5 (*74*).<br>$k_{target\text{-}temp} = k_{ref\text{-}temp} * Q10^{(Ttarget\text{-}Tref) / 10ºC}$

REFERENCES AND NOTES

1. T. Iida, M. Furutani, T. Iwabuchi, T. Maruyama, Gene for a cyclophilin-type peptidyl-prolyl cis-trans isomerase from a halophilic archaeum, Halobacterium cutirubrum. *Gene* **204**, 139–144 (1997).

2. M. A. Stamnes, S. L. Rutherford, C. S. Zuker, Cyclophilins: A new family of proteins involved in intracellular folding. *Trends Cell Biol.* **2**, 272–276 (1992).

3. A. Clery, M. Blatter, F. H. Allain, RNA recognition motifs: Boring? Not quite. *Current Opin. Struct. Biol.* **18**, 290–298 (2008).

4. R. A. Hom, P. Y. Chang, S. Roy, C. A. Musselman, K. C. Glass, A. I. Selezneva, O. Gozani, R. F. Ismagilov, M. L. Cleary, T. G. Kutateladze, Molecular mechanism of MLL PHD3 and RNA recognition by the Cyp33 RRM domain. *J. Mol. Biol.* **400**, 145–154 (2010).

5. M. Anderson, K. Fair, S. Amero, S. Nelson, P. Harte, M. Diaz, A new family of cyclophilins with an RNA recognition motif that interact with members of the trx/MLL protein family in *Drosophila* and human cells. *Dev. Genes Evol.* **212**, 107–113 (2002).

6. Y. Wang, R. Han, W. Zhang, Y. Yuan, X. Zhang, Y. Long, H. Mi, Human CyP33 binds specifically to mRNA and binding stimulates PPIase activity of hCyP33. *FEBS Lett.* **582**, 835–839 (2008).

7. H. Mi, O. Kops, E. Zimmermann, A. Jaschke, M. Tropschug, A nuclear RNA-binding cyclophilin in human T cells. *FEBS Lett.* **398**, 201–205 (1996).

8. K. Fair, M. Anderson, E. Bulanova, H. Mi, M. Tropschug, M. O. Diaz, Protein interactions of the MLL PHD fingers modulate MLL target gene regulation in human cells. *Mol. Cell. Biol.* **21**, 3589–3597 (2001).

9. C. D. Jude, L. Climer, D. Xu, E. Artinger, J. K. Fisher, P. Ernst, Unique and independent roles for MLL in adult hematopoietic stem cells and progenitors. *Cell Stem Cell* **1**, 324–337 (2007).

10. B. D. Yu, R. D. Hanson, J. L. Hess, S. E. Horning, S. J. Korsmeyer, MLL, a mammalian trithorax-group gene, functions as a transcriptional maintenance factor in morphogenesis. *Proc. Natl. Acad. Sci. U.S.A.* **95**, 10632–10636 (1998).

11. S. Ziemin-van der Poel, N. R. McCabe, H. J. Gill, R. Espinosa III, Y. Patel, A. Harden, P. Rubinelli, S. D. Smith, M. M. LeBeau, J. D. Rowley, Identification of a gene, MLL, that spans the breakpoint in 11q23 translocations associated with human leukemias. *Proc. Natl. Acad. Sci. U.S.A.* **88**, 10735–10739 (1991).

12. P. Y. Chang, R. A. Hom, C. A. Musselman, L. Zhu, A. Kuo, O. Gozani, T. G. Kutateladze, M. L. Cleary, Binding of the MLL PHD3 finger to histone H3K4me3 is required for MLL-dependent gene transcription. *J. Mol. Biol.* **400**, 137–144 (2010).

13. Z. Wang, J. Song, T. A. Milne, G. G. Wang, H. Li, C. D. Allis, D. J. Patel, Pro isomerization in MLL1 PHD3-bromo cassette connects H3K4me readout to CyP33 and HDAC-mediated repression. *Cell* **141**, 1183–1194 (2010).

14. Y. Li, J. Han, Y. Zhang, F. Cao, Z. Liu, S. Li, J. Wu, C. Hu, Y. Wang, J. Shuai, J. Chen, L. Cao, D. Li, P. Shi, C. Tian, J. Zhang, Y. Dou, G. Li, Y. Chen, M. Lei, Structural basis for activity regulation of MLL family methyltransferases. *Nature* **530**, 447–452 (2016).

15. J. J.-D. Hsieh, E. H.-Y. Cheng, S. J. Korsmeyer, Taspase1: A threonine aspartase required for cleavage of MLL and proper HOX gene expression. *Cell* **115**, 293–303 (2003).

16. J. J. Hsieh, P. Ernst, H. Erdjument-Bromage, P. Tempst, S. J. Korsmeyer, Proteolytic cleavage of MLL generates a complex of N- and C-terminal fragments that confers protein stability and subnuclear localization. *Mol. Cell. Biol.* **23**, 186–194 (2003).

17. M. S. Cosgrove, A. Patel, Mixed lineage leukemia: A structure-function perspective of the MLL1 protein. *FEBS J.* **277**, 1832–1842 (2010).

18. W. D. Jones, D. Dafou, M. McEntagart, W. J. Woollard, F. V. Elmslie, M. Holder-Espinasse, M. Irving, A. K. Saggar, S. Smithson, R. C. Trembath, C. Deshpande, M. A. Simpson, De

novo mutations in MLL cause Wiedemann-Steiner syndrome. *Am. J. Hum. Genet.* **91**, 358–364 (2012).

19. A. G. Muntean, J. L. Hess, The pathogenesis of mixed-lineage leukemia. *Annu. Rev. Pathol.* **7**, 283–301 (2012).

20. M. Mohan, C. Lin, E. Guest, A. Shilatifard, Licensed to elongate: A molecular mechanism for MLL-based leukaemogenesis. *Nat. Rev. Cancer* **10**, 721–728 (2010).

21. H. Liu, E. H. Cheng, J. J. Hsieh, MLL fusions: Pathways to leukemia. *Cancer Biol. Ther.* **8**, 1204–1211 (2009).

22. A. Daser, T. H. Rabbitts, The versatile mixed lineage leukaemia gene MLL and its many associations in leukaemogenesis. *Semin. Cancer Biol.* **15**, 175–188 (2005).

23. A. V. Krivtsov, S. A. Armstrong, MLL translocations, histone modifications and leukaemia stem-cell development. *Nat. Rev. Cancer* **7**, 823–833 (2007).

24. C. Meyer, T. Burmeister, D. Gröger, G. Tsaur, L. Fechina, A. Renneville, R. Sutton, N. C. Venn, M. Emerenciano, M. S. Pombo-de-Oliveira, C. Barbieri Blunck, B. Almeida Lopes, J. Zuna, J. Trka, P. Ballerini, H. Lapillonne, M. de Braekeleer, G. Cazzaniga, L. Corral Abascal, V. H. J. van der Velden, E. Delabesse, T. S. Park, S. H. Oh, M. L. M. Silva, T. Lund-Aho, V. Juvonen, A. S. Moore, O. Heidenreich, J. Vormoor, E. Zerkalenkova, Y. Olshanskaya, C. Bueno, P. Menendez, A. Teigler-Schlegel, U. zur Stadt, J. Lentes, G. Göhring, A. Kustanovich, O. Aleinikova, B. W. Schäfer, S. Kubetzko, H. O. Madsen, B. Gruhn, X. Duarte, P. Gameiro, E. Lippert, A. Bidet, J. M. Cayuela, E. Clappier, C. N. Alonso, C. M. Zwaan, M. M. van den Heuvel-Eibrink, S. Izraeli, L. Trakhtenbrot, P. Archer, J. Hancock, A. Möricke, J. Alten, M. Schrappe, M. Stanulla, S. Strehl, A. Attarbaschi, M. Dworzak, O. A. Haas, R. Panzer-Grümayer, L. Sedék, T. Szczepański, A. Caye, L. Suarez, H. Cavé, R. Marschalek, The MLL recombinome of acute leukemias in 2017. *Leukemia* **32**, 273–284 (2018).

25. R. K. Slany, The molecular biology of mixed lineage leukemia. *Haematologica* **94**, 984–993 (2009).

26. A. G. Muntean, D. Giannola, A. M. Udager, J. L. Hess, The PHD fingers of MLL block MLL fusion protein-mediated transformation. *Blood* **112**, 4690–4693 (2008).

27. J. Chen, D. A. Santillan, M. Koonce, W. Wei, R. Luo, M. J. Thirman, N. J. Zeleznik-le, M. O. Diaz, Loss of MLL PHD finger 3 is necessary for MLL-ENL-induced hematopoietic stem cell immortalization. *Cancer Res.* **68**, 6199–6207 (2008).

28. S. Park, U. Osmers, G. Raman, R. H. Schwantes, M. O. Diaz, J. H. Bushweller, The PHD3 domain of MLL acts as a CYP33-regulated switch between MLL-mediated activation and repression. *Biochemistry* **49**, 6576–6586 (2010).

29. M. Wang, M. W. Mok, H. Harper, W. H. Lee, J. Min, S. Knapp, U. Oppermann, B. Marsden, M. Schapira, Structural genomics of histone tail recognition. *Bioinformatics* **26**, 2629–2630 (2010).

30. P. L. Hsu, H. Li, H. T. Lau, C. Leonen, A. Dhall, S. E. Ong, C. Chatterjee, N. Zheng, Crystal structure of the COMPASS H3K4 methyltransferase catalytic module. *Cell* **174**, 1106–1116.e9 (2018).

31. Q. Qu, Y. H. Takahashi, Y. Yang, H. Hu, Y. Zhang, J. S. Brunzelle, J. F. Couture, A. Shilatifard, G. Skiniotis, Structure and conformational dynamics of a COMPASS histone H3K4 methyltransferase complex. *Cell* **174**, 1117–1126.e12 (2018).

32. J. Schneider, A. Wood, J. S. Lee, R. Schuster, J. Dueker, C. Maguire, S. K. Swanson, L. Florens, M. P. Washburn, A. Shilatifard, Molecular regulation of histone H3 trimethylation by COMPASS and the regulation of gene expression. *Mol. Cell* **19**, 849–856 (2005).

33. G. Raman, thesis, Loyola University Chicago (2013).

34. X. Wang, S. Arai, X. Song, D. Reichart, K. du, G. Pascual, P. Tempst, M. G. Rosenfeld, C. K. Glass, R. Kurokawa, Induced ncRNAs allosterically modify RNA-binding proteins in cis to inhibit transcription. *Nature* **454**, 126–130 (2008).

35. N. R. Lloyd, D. S. Wuttke, Cyp33 binds AU-rich RNA motifs via an extended interface that competitively disrupts the gene repressive Cyp33-MLL1 interaction in vitro. *PLOS ONE* **16**, e0237956 (2021).

36. J. A. Solanki, thesis, Loyola University Chicago, Chicago (2011).

37. Z. B. Xia, M. Anderson, M. O. Diaz, N. J. Zeleznik-Le, MLL repression domain interacts with histone deacetylases, the polycomb group proteins HPC2 and BMI-1, and the corepressor C-terminal-binding protein. *Proc. Natl. Acad. Sci. U.S.A.* **100**, 8342–8347 (2003).

38. Y. Shen, F. Delaglio, G. Cornilescu, A. Bax, TALOS+: A hybrid method for predicting protein backbone torsion angles from NMR chemical shifts. *J. Biomol. NMR* **44**, 213–223 (2009).

39. F. H.-T. Allain, C. C. Gubser, P. W. A. Howe, K. Nagai, D. Neuhaus, G. Varani, Specificity of ribonucleoprotein interaction determined by RNA folding during complex formation. *Nature* **380**, 646–650 (1996).

40. C. Maris, S. Jayne, F. F. Damberger, I. Beusch, G. Dorn, S. Ravindranathan, F. H. T. Allain, A transient α-helix in the N-terminal RNA recognition motif of polypyrimidine tract binding protein senses RNA secondary structure. *Nucleic Acids Res.* **48**, 4521–4537 (2020).

41. K. C. Wang, Y. W. Yang, B. Liu, A. Sanyal, R. Corces-Zimmerman, Y. Chen, B. R. Lajoie, A. Protacio, R. A. Flynn, R. A. Gupta, J. Wysocka, M. Lei, J. Dekker, J. A. Helms, H. Y. Chang, A long noncoding RNA maintains active chromatin to coordinate homeotic gene expression. *Nature* **472**, 120–124 (2011).

42. R. Maldonado, U. Schwartz, E. Silberhorn, G. Längst, Nucleosomes stabilize ssRNA-dsDNA triple helices in human cells. *Mol. Cell* **73**, 1243–1254.e6 (2019).

43. R. Dueva, K. Akopyan, C. Pederiva, D. Trevisan, S. Dhanjal, A. Lindqvist, M. Farnebo, Neutralization of the positive charges on histone tails by RNA promotes an open chromatin structure. *Cell Chem. Biol.* **26**, 1436–1449.e5 (2019).

44. A. Bhan, P. Deb, N. Shihabeddin, K. I. Ansari, M. Brotto, S. S. Mandal, Histone methylase MLL1 coordinates with HIF and regulate lncRNA HOTAIR expression under hypoxia. *Gene* **629**, 16–28 (2017).

45. P. Grote, L. Wittler, D. Hendrix, F. Koch, S. Währisch, A. Beisaw, K. Macura, G. Bläss, M. Kellis, M. Werber, B. G. Herrmann, The tissue-specific lncRNA Fendrr is an essential regulator of heart and body wall development in the mouse. *Dev. Cell* **24**, 206–214 (2013).

46. C. Deng, Y. Li, L. Zhou, J. Cho, B. Patel, N. Terada, Y. Li, J. Bungert, Y. Qiu, S. Huang, HoxBlinc RNA recruits Set1/MLL complexes to activate hox gene expression patterns and mesoderm lineage development. *Cell Rep.* **14**, 103–114 (2016).

47. Y. W. Yang, R. A. Flynn, Y. Chen, K. Qu, B. Wan, K. C. Wang, M. Lei, H. Y. Chang, Essential role of lncRNA binding for WDR5 maintenance of active chromatin and embryonic stem cell pluripotency. *eLife* **3**, e02046 (2014).

48. V. Kasinath, M. Faini, S. Poepsel, D. Reif, X. A. Feng, G. Stjepanovic, R. Aebersold, E. Nogales, Structures of human PRC2 with its cofactors AEBP2 and JARID2. *Science* **359**, 940–944 (2018).

49. H. Xue, T. Yao, M. Cao, G. Zhu, Y. Li, G. Yuan, Y. Chen, M. Lei, J. Huang, Structural basis of nucleosome recognition and modification by MLL methyltransferases. *Nature* **573**, 445–449 (2019).

50. C. Wei, R. Xiao, L. Chen, H. Cui, Y. Zhou, Y. Xue, J. Hu, B. Zhou, T. Tsutsui, J. Qiu, H. Li, L. Tang, X. D. Fu, RBFox2 binds nascent RNA to globally regulate polycomb complex 2 targeting in mammalian genomes. *Mol. Cell* **62**, 875–889 (2016).

51. D. Holoch, D. Moazed, RNA-mediated epigenetic regulation of gene expression. *Nat. Rev. Genet.* **16**, 71–84 (2015).

52. F. H. Niesen, H. Berglund, M. Vedadi, The use of differential scanning fluorimetry to detect ligand interactions that promote protein stability. *Nat. Protoc.* **2**, 2212–2221 (2007).

53. J. Cavanagh, M. Akke, May the driving force be with you—Whatever it is. *Nat. Struct. Biol.* **7**, 11–13 (2000).

54. M. Sattler, J. Schleucher, C. Griesinger, Heteronuclear multidimensional NMR experiments for the structure determination of proteins in solution employing pulsed field gradients. *Prog. Nucl. Magn. Reson. Spectrosc.* **34**, 93–158 (1999).

55. L. E. Kay, D. A. Torchia, A. Bax, Backbone dynamics of proteins as studied by 15N inverse detected heteronuclear NMR spectroscopy: Application to staphylococcal nuclease. *Biochemistry* **28**, 8972–8979 (1989).

56. P. Guntert, Automated NMR structure calculation with CYANA. *Methods Mol. Biol.* **278**, 353–378 (2004).

57. D. A. Pearlman, D. A. Case, J. W. Caldwell, W. S. Ross, T. E. Cheatham III, S. DeBolt, D. Ferguson, G. Seibel, P. Kollman, AMBER, a package of computer programs for applying molecular mechanics, normal mode analysis, molecular dynamics and free energy calculations to simulate the structural and energetic properties of molecules. *Comput. Phys. Commun.* **91**, 1–41 (1995).

58. K. Lindorff-Larsen, S. Piana, K. Palmo, P. Maragakis, J. L. Klepeis, R. O. Dror, D. E. Shaw, Improved side-chain torsion potentials for the Amber ff99SB protein force field. *Proteins* **78**, 1950–1958 (2010).

59. D. Bashford, D. A. Case, Generalized born models of macromolecular solvation effects. *Annu. Rev. Phys. Chem.* **51**, 129–152 (2000).

60. T. Darden, D. York, L. Pedersen, Particle mesh Ewald: AnN·log(N) method for Ewald sums in large systems. *J. Chem. Phys.* **98**, 10089–10092 (1993).

61. J.-P. Ryckaert, G. Ciccotti, H. J. C. Berendsen, Numerical integration of the cartesian equations of motion of a system with constraints: Molecular dynamics of n-alkanes. *J. Comput. Phys.* **23**, 327–341 (1977).

62. R. A. Laskowski, J. A. Rullmannn, M. W. MacArthur, R. Kaptein, J. M. Thornton, AQUA and PROCHECK-NMR: Programs for checking the quality of protein structures solved by NMR. *J. Biomol. NMR* **8**, 477–486 (1996).

63. J. Sambrook, D. Russel, in *Molecular Cloning: A Laboratory Manual* (Cold Spring Harbor Laboratory Press, USA, ed. 3, 2000), vol. 1–3.

64. E. Sakashita, H. Sakamoto, Characterization of RNA binding specificity of the *Drosophila* sex-lethal protein by in vitro ligand selection. *Nucleic Acids Res.* **22**, 4082–4086 (1994).

65. L. A. Harris, J. S. Hogg, J.-J. Tapia, J. A. P. Sekar, S. Gupta, I. Korsunsky, A. Arora, D. Barua, R. P. Sheehan, J. R. Faeder, BioNetGen 2.2: Advances in rule-based modeling. *Bioinformatics* **32**, 3366–3368 (2016).

66. A. M. Smith, W. Xu, Y. Sun, J. R. Faeder, G. E. Marai, RuleBender: Integrated modeling, simulation and visualization for rule-based intracellular biochemistry. *BMC Bioinformatics* **13** (Suppl. 8), S3 (2012).

67. J. L. Kofron, P. Kuzmic, V. Kishore, E. Colon-Bonilla, D. H. Rich, Determination of kinetic constants for peptidyl prolyl cis-trans isomerases by an improved spectrophotometric assay. *Biochemistry* **30**, 6127–6134 (1991).

68. D. S. Cabianca, V. Casa, B. Bodega, A. Xynos, E. Ginelli, Y. Tanaka, D. Gabellini, A long ncRNA links copy number variation to a polycomb/trithorax epigenetic switch in FSHD muscular dystrophy. *Cell* **149**, 819–831 (2012).

69. Y. Jeon, J. T. Lee, YY1 tethers Xist RNA to the inactive X nucleation center. *Cell* **146**, 119–133 (2011).

70. S. Qin, X. Pang, H. X. Zhou, Automated prediction of protein association rate constants. *Structure* **19**, 1744–1751 (2011).

71. M. B. R. Hołyst, K. Burdzy, G. Góralski, L. Bocquet, Reduction of dimensionality in a diffusion search process and kinetics of gene expression. *Physica A***7**, 71–82 (2000).

72. T. Pan, T. Sosnick, RNA folding during transcription. *Annu. Rev. Biophys. Biomol. Struct.* **35**, 161–175 (2006).

73. R. K. Harrison, R. L. Stein, Substrate specificities of the peptidyl prolyl cis-trans isomerase activities of cyclophilin and FK-506 binding protein: Evidence for the existence of a family of distinct enzymes. *Biochemistry* **29**, 3813–3816 (1990).

74. B. A. Reyes, J. S. Pendergast, S. Yamazaki, Mammalian peripheral circadian oscillators are temperature compensated. *J. Biol. Rhythms* **23**, 95–98 (2008).
